# Discovery of conformation constrained tetracyclic compounds as potent chitinase *Of*Chi-h inhibitors with a novel binding mode

**DOI:** 10.1080/14756366.2025.2528056

**Published:** 2025-07-07

**Authors:** Pengtao Yuan, Xi Jiang, Xintong Ni, Xusheng Shao, Xuhong Qian, Qing Yang

**Affiliations:** a Shenzhen Branch, Guangdong Laboratory for Lingnan Modern Agriculture, Key Laboratory of Synthetic Biology, Ministry of Agriculture and Rural Affairs, Agricultural Genomics Institute at Shenzhen, Chinese Academy of Agricultural Sciences, Shenzhen, China; b State Key Laboratory for Biology of Plant Diseases and Insect Pests, Institute of Plant Protection, Chinese Academy of Agricultural Sciences, Beijing, China; c College of Life and Health, Dalian University, Dalian, China; d Shanghai Key Laboratory of Chemical Biology, School of Pharmacy, East China University of Science and Technology, Shanghai, China; e School of Chemistry and Molecular Engineering, East China Normal University, Shanghai, China

**Keywords:** Chitinase, inhibitor, conformation restriction, inhibitory mechanism

## Abstract

Chitinase h (Chi-h) has been identified as a promising pesticide target due to its exclusive distribution in lepidopteran insects and its essential role in the moulting processes. In this study, we leverage *Of*Chi-h from destructive agricultural pest *Ostrinia furnacalis* (Asian corn borer) as a model target to identify novel chitinase inhibitors. A conformational restriction approach was employed to design a series of novel *Of*Chi-h inhibitors. Among these, compound **6a** showed the highest inhibitory activity against *Of*Chi-h, with a *K_i_* value of 58 nM. Molecular docking analysis suggested that **6a** tightly bound to three subsites (-3 to −1) of *Of*Chi-h. The binding mode is further confirmed by the co-crystallization data of **6a** with the *Sm*ChiA, a bacterial homologue of *Of*Chi-h, at a resolution of 1.8 Å. This research presents a novel approach for the development of highly potent insect chitinase inhibitors, offering potential tools for effective pest control.

## GRAPHIC ABSTRACT

**Figure:**
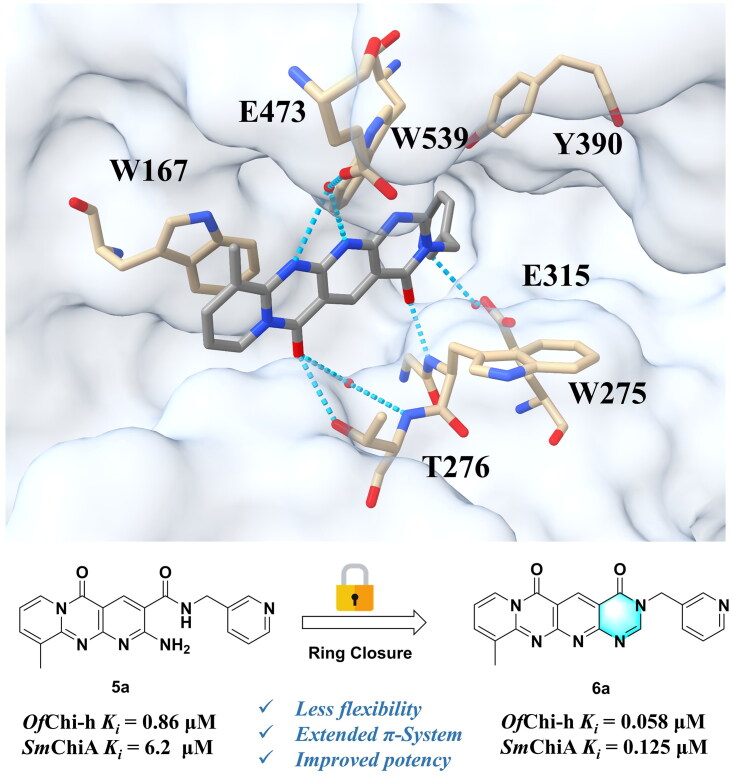

## Introduction

Chitinase (EC 3.2.1.14) is an enzyme that hydrolyses β-1,4-glycosidic bonds in chitin and chitooligosaccharides. The loss of chitinase enzymatic activity in insects results in severe exoskeletal defects and lethality at all developmental stages, as chitin is an important structural component of insect cuticle^1^. Chitinase-h(Chi-h) is a lepidoptera-exclusive insect chitinase that is absent in most beneficial insects including parasitic wasps and bees. Chi-h is thought to be acquired through horizontal gene transfer from bacteria^2^ and it shows significantly lower sequence similarity to other insect chitinases. Moreover, the structure of Chi-h is significantly different from that of human and other insect chitinases^3^. Thus, Chi-h is a promising target, and its inhibitors hold potential for eco-friendly pesticide development, contributing to sustainable agriculture^4^.

**Figure 1.**
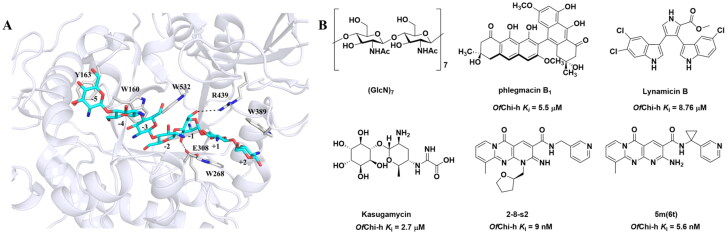
Binding clef of *Of*Chi-h and its inhibitors. (a) *Of*Chi-h in complex with (GlcN)_7_ (PDB: 5GQB) and (b) representative chitinase *Of*Chi-h inhibitors.

The only crystal structure of Chi-h is derived from *Ostrinia furnacalis* (*Of*Chi-h), thereby establishing it as an important model for the development of Chi-h inhibitors^1^^,^^3–5^. *Of*Chi-h features an elongated, asymmetric substrate-binding cleft containing seven distinct sugar-binding subsites, with ­multiple aromatic residues strategically positioned along the groove’s architecture. The co-crystal complex of *Of*Chi-h**-**(GlcN)_7_ revealed that seven subsites, spanning from −5 to +2, are present in the binding cleft^3^ (Figure 1(a)). The sugar-binding subsites were named according to Davies et al.^6^ where subsite -n represents the non-reducing end, subsite + n represents the reducing end, and enzymatic cleavage occurs between the −1 and the +1 subsites. To date, several *Of*Chi-h inhibitors have been reported, including chitoheptaose (**(GlcN)_7_**)^3^, the microbial secondary metabolite phlegmacin **B1**^7^, the marine natural product Lynamicin B^8^, the aminoglycoside antibiotic Kasugamycin^9^, compound **2–8-s2**^10^, compound **5 m** (also named as **6t** in our previous report)^11^ and berberine along with its derivatives^12^^,^^13^. Several multitarget inhibitors of insect chitinolytic enzymes have also been reported, including Shikonin, Wogonin^14^, 3,5-Di-O-caffeoylquinic acid and γ-mangostin^15^ Rhein and its dimeric analogue Sennidin B^16^, Butenolide derivatives^17^^,^^18^ and guanidine-based inhibitors^19–22^.

Conformational restriction is a well-known strategy in drug and pesticide development^23^^,^^24^, which could be achieved by the enhancement of steric effects^25^, intramolecular hydrogen bonding^26^, ion pairing^27^, π–π stacking, incorporation of cyclic elements^28^, or fusion of adjacent rings. Compared with flexible ligands, similarly restrained ligands can form analogous pairwise interactions with the target while sacrificing fewer conformational degrees of freedom during complex formation, thereby resulting in a lower entropic penalty^29^ and improved binding affinity. In addition, conformational restriction by introducing fused rings offers potential advantages, such as reducing degradation and increasing selectivity towards certain receptor subtypes^30^.

GH18 chitinases possess surface-exposed aromatic residues in the binding cleft, which interact with sugars by a combination of hydrogen bonding and hydrophobic stacking interactions and have been shown to be essential for substrate binding. Although all the GH18 chitinases use the same catalytic mechanism, they exhibit variations in the shape of their substrate-binding cleft. Thus, rigid structures with fused rings may selectively inhibit certain types of chitinases. In our previous study, we identified **5 m**^11^ as a potent *Of*Chi-h inhibitor by introducing steric groups to restrain the conformation of **5a** (Figure 1(b)). In this work, we reported the discovery of a novel rigid tetracyclic scaffold for *Of*Chi-h inhibitors by ring closure to fix the conformation, with the expectation that it will enhance both inhibitory activity and selectivity. Among the designed compounds, compound **6a** showed the highest inhibitory activity against *Of*Chi-h with a *K_i_* value of 58 nM. Furthermore, the structural analysis and docking results revealed that the tetracyclic scaffold binds to different sites within the chitinase, which will guide future chitinase inhibitors design.

## Results and discussion

### Rational design of a novel tetracyclic core

**Figure 2.**
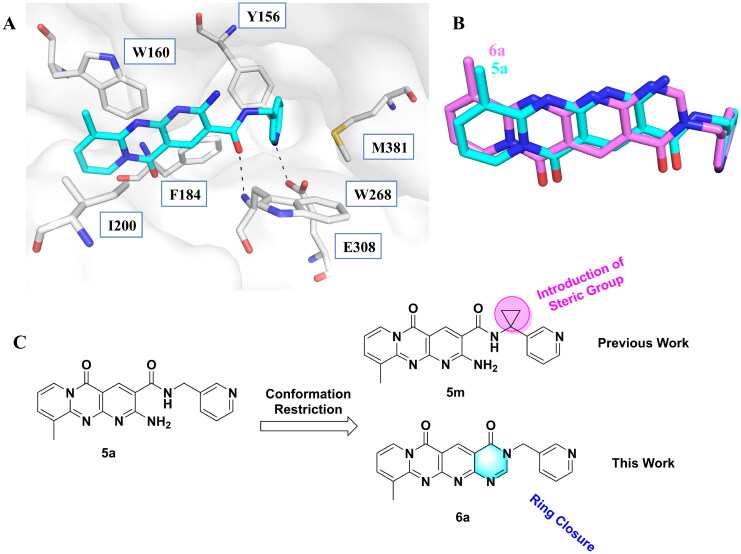
Design strategy of novel tetracyclic chitinase inhibitors. (a) Predicted binding mode of compound **5a** with *Of*Chi-h; (b) superimposition of compound **5a** and compound **6a**; (c) novel conformation restriction strategy by ring closure.

As previously reported, compound **5a** was involved in several key interactions with residues in the active site of the chitinase as revealed by its X-ray cocrystal structure^11^. For example, the pyridopyrimidine moiety of compound **5a** stacked with Trp97 and Trp220 at +1 and +2 subsites, respectively, and the 3-pyridyl nitrogen formed a hydrogen bond with the key catalytic residue Glu144. Interestingly, molecular docking indicated that **5a** binds to *Of*Chi-h in a different way. It is predicted to bind in the −3, −2 and −1 subsites of *Of*Chi-h, while still forming a hydrogen bond with the key catalytic residue Glu308 (Figure 2(a)). In addition, the carbonyl oxygen atom of the amide group forms a hydrogen bond with the backbone of Trp268. Therefore, we designed a tetracyclic scaffold, compound **6a,** with fewer rotatable bonds to lock it covalently into an active conformation, thereby improving potency. As shown by the superimposition of **5a** and **6a** (Figure 2(b)), the rigid tetracyclic core of **6a** positions the carbonyl oxygen atom and the pyridine ring nearly exactly where they must be to maintain the active conformation of **5a**. With this information in hand, we proceeded to synthesis **6a** to test our hypothesis that conformation restriction improves potency.

### Chemistry

**Scheme 1.**
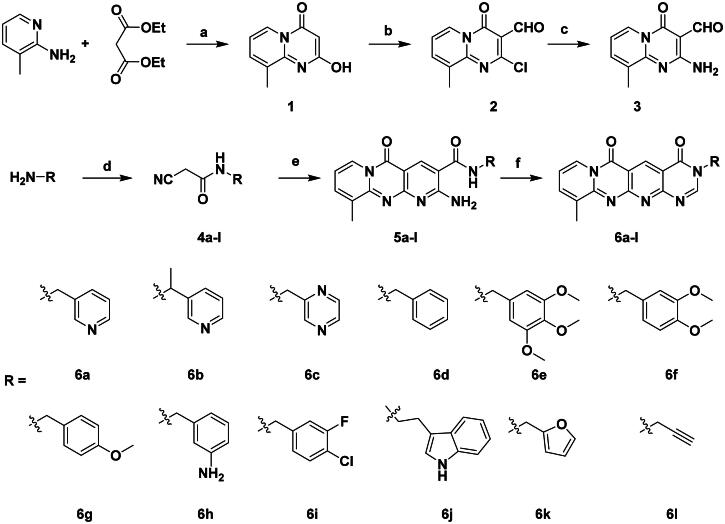
Synthesis of target compounds **6a-l.** Reagents and conditions: (a). neat, 110 °C; (b). POCl_3_, DMF, 0 °C-80 °C; (c). aqueous ammonia, EtOH, 70 °C; (d). methyl cyanoacetate, r.t. (e). NaOH/EtOH, 70 °C; (f). DMFDMA, neat, 110 °C.

The target compounds **6a–6l** were synthesised following the synthetic route depicted in Scheme 1. The starting material, 2-hydroxy-9-methyl-4H-pyrido[1,2-a]pyrimidin-4-one (compound **1**), was prepared via a condensation reaction between 2-amino-3-picoline and diethyl malonate at 110 °C. Treatment of compound **1** with phosphorus oxychloride (POCl_3_) in DMF afforded 2-chloro-9-methyl-4-oxo-4H-pyrido[1,2-a]pyrimidine-3-carbaldehyde (compound **2**), which was then reacted with excess aqueous ammonia in ethanol at 70 °C to give the key intermediate 2-amino-9-methyl-4-oxo-4H-pyrido[1,2-a]pyrimidine-3-carbaldehyde (compound **3**). The cyanoacetamide derivatives **4a–4l** were synthesised by the condensation of methyl cyanoacetate with the corresponding amines under neat conditions at room temperature. Subsequent condensation of intermediate **3** with cyanoacetamides **4a–4l** in the presence of sodium hydroxide furnished compounds **5a–5l** via a modified Friedländer reaction. Finally, treatment of **5a–5l** with 1,1-dimethoxy-*N,N*-dimethylmethanamine (DMFDMA) under neat conditions at 110 °C yielded the desired tetracyclic target compounds **6a–6l**. All the target compounds were confirmed by NMR spectroscopy and HRMS.

### Structure-activity relationship analysis

**Figure 3.**
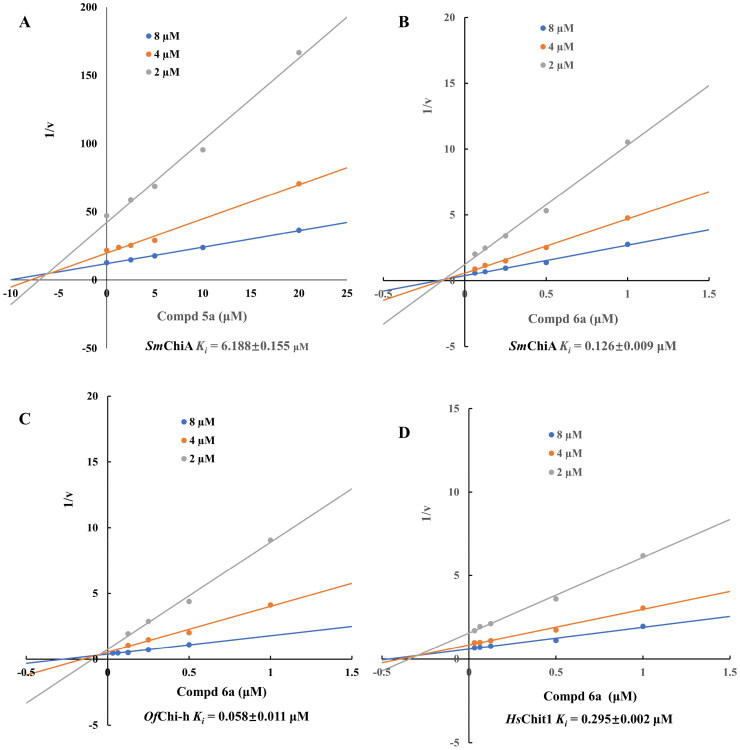
Representative Dixon plots for inhibition chitinase *Sm*Chi-A by **5a** (a), and chitinase *Sm*Chi-A (b), *Of*Chi-h (c) and *Hs*Chit1 (d) by **6a**. The trend lines represent three substrate concentrations.

Originally, we sought to condense **5a** with DMFDMA to afford **6a**. As expected, **6a** was obtained and tested against *Of*Chi-h. To our delight, **6a** showed a significant increase in inhibitory activity against *Of*Chi-h, with a *K*_i_ value of 0.058 μM (Table 1 and Figure 3) compared to that of **5a**^11^ (an approximately 15-fold improvement). The inspiring result motivated further modifications of **6a**. Initially, a methyl group was introduced to the side chain of the pyridine to afford **6b** (Figure S1). However, this modification unexpectedly resulted in a sharp decrease in inhibitory activity. Due to unsuccessful condensation reactions when larger substituents were introduced on the pyridine side chain, pyridine was replaced by pyrazine to produce compound **6c**, in an effort to facilitate additional interactions with *Of*Chi-h. However, **6c** showed similar inhibitory activity to **6b** (Figure S2). Compound **6d**, bearing a phenyl substituent, exhibited further decreased inhibitory activity, with a *K_i_* value of 1.419 μM (Figure S3). Though the introduction of fluorine and chlorine (**6i**) to the phenyl group failed to improve activity, the incorporation of an amino group (**6h**) and a methoxyl group (**6 g**) increased activity related to **6d** (Figures S4 and S5). Surprisingly, the installation of a second methoxyl group (**6f**) and a third methoxyl group (**6e**) further enhanced the inhibitory activity against *Of*Chi-h. **6e** showed comparable activity to **6a**, with a *K_i_* value of 0.065 μM (Figure S3). Other attempts to substitute the pyridine group (**6j-6l**) also resulted in decreased activity, indicating that the pyridine group may interact with *Of*Chi-h (Figures S4 and S5). We also tried to fuse **5 m** to obtain a tetracyclic scaffold, as it showed the most potent activity in our previous study. However, the reaction is sensitive to the side chain of the amide, and **5 m** failed to condensed into the corresponding tetracyclic structure due to the presence of a cyclopropyl group—a similar phenomenon was also observed by Wang^31^. In addition, the most potent compound **6a** also displayed good inhibition activity against *Sm*ChiA (a bacterial homology of *Of*Chi-h from *Serratia marcescens*) and *Hs*Chit1 (a chitinase from *Homo sapiens*), with a *K_i_* value of 0.126 μM and 0.295 μM, respectively (Figure 3(b,d)).

**Figure 4.**
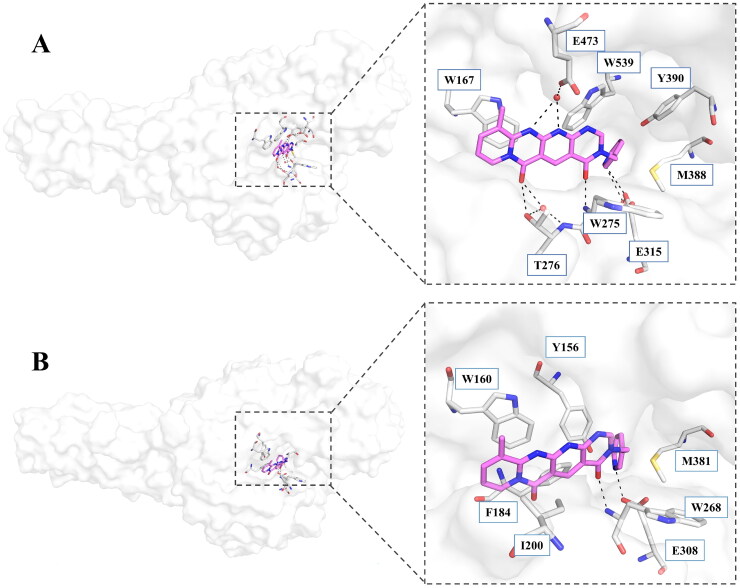
Binding mode of **6a** with *Sm*ChiA and *Of*Chi-h. A. Binding mode of **6a** with *Sm*ChiA revealed by co-crystal complex structure; B. Binding mode of **6a** with *Of*Chi-h revealed by molecular docking. **6a** is shown in violet sticks, the key residues involved in inhibitor binding are shown in gray sticks, hydrogen bonds are shown as black dashed lines and water molecules are shown as red spheres.

**Figure 5.**
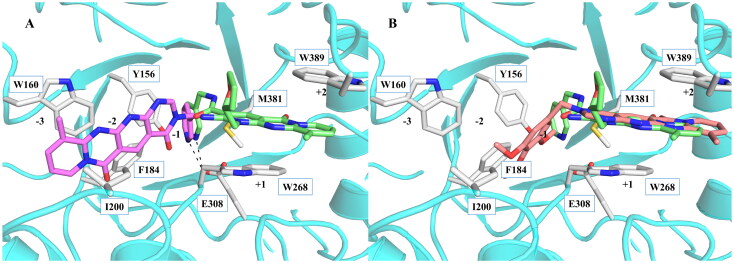
Superposition of **6a** (a) and **6e** (b) with **2–8-s2** in the binding site of *Of*Chi-h. **6a** is shown in violet sticks, **6e** was shown in red sticks, **2–8-s2** was shown in light green sticks and the key residues involved in inhibitor binding are shown in gray sticks, hydrogen bonds are shown as black dashed lines.

**Table 1.** Inhibition activity of **6a-6l** against *Sm*ChiA and *Of*Chi-h at 10 μM.

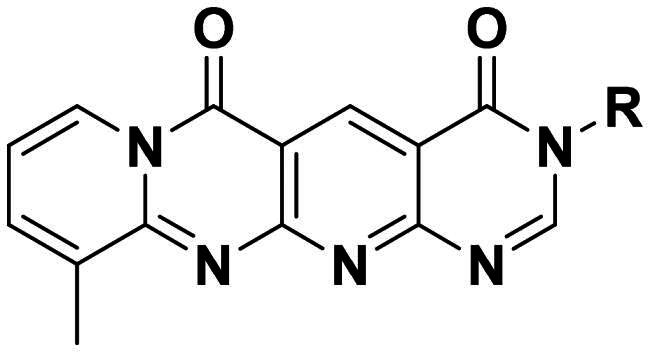				
Compd	R	*Sm*ChiA	*Of*Chi-h	
		10 μM (%)	10 μM (%)	*K_i_* (μM)
**6a**	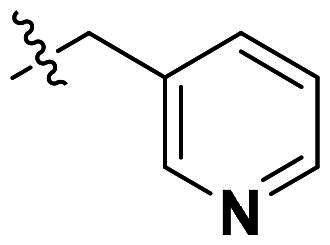	98.5 ± 0.2%	99.3 ± 0.2%	0.058
**6b**	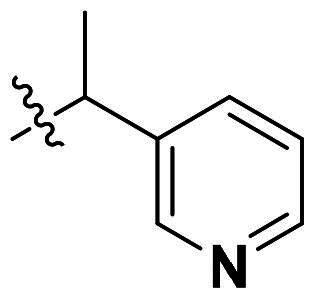	88.7 ± 2.2%	96.8 ± 0.1%	0.215
**6c**	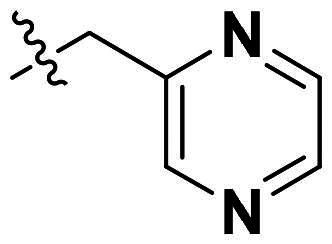	91.5 ± 0.8%	95.3 ± 0.5%	0.294
**6d**	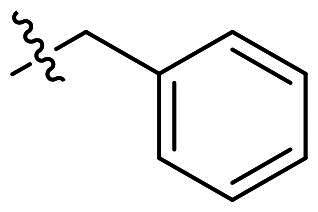	32.0 ± 1.4%	72.5 ± 2.4%	1.419
**6e**	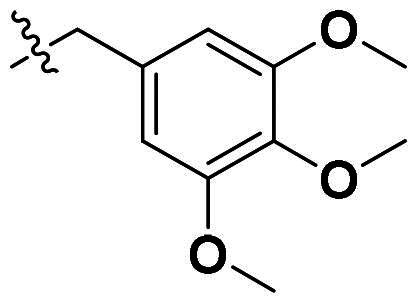	28.2 ± 4.8%	96.2 ± 0.3%	0.065
**6f**	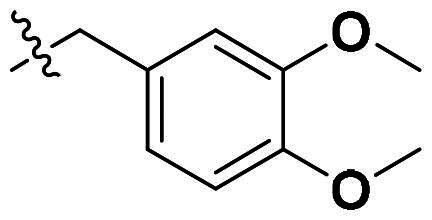	34.2 ± 2.5%	69.3 ± 4.4%	0.747
**6g**	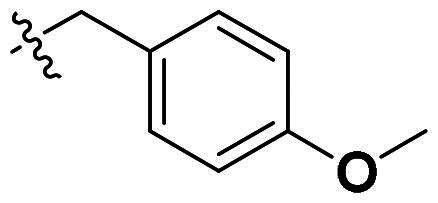	30.3 ± 2.7%	74.0 ± 2.0%	1.032
**6h**	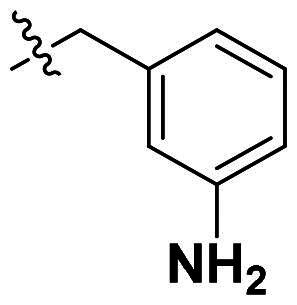	37.9 ± 0.4%	89.0 ± 1.5%	0.276
**6i**	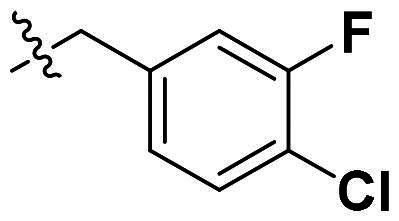	31.7 ± 0.2%	42.9 ± 1.0%	2.889
**6j**	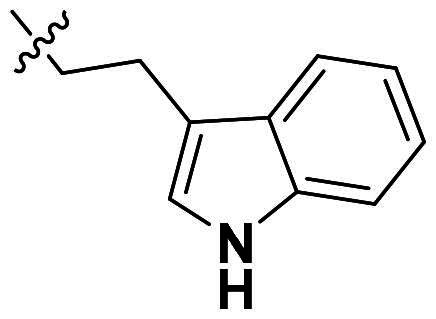	42.4 ± 4.1%	49.6 ± 2.2%	0.551
**6k**	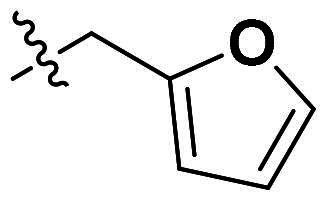	47.5 ± 1.2%	86.8 ± 0.8%	1.509
**6l**	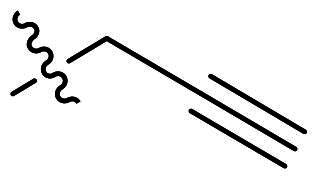	37.5 ± 2.2%	81.0 ± 0.8%	1.338
**5a** ^11^	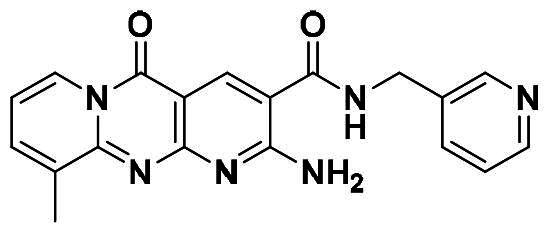	–	–	0.86

### Elucidation of binding modes to OfChi-h

To investigate the binding mode of fused tetracyclic scaffold and to validate our hypothesis, we tried to obtain the crystal structure of *Of*Chi-h and *Sm*ChiA in complex with the inhibitor **6a.** However, only co-crystal structure *Sm*ChiA-6a was successfully obtained and refined. In this structure, compound **6a** is bound at the −3, −2 and −1 subsites of *Sm*ChiA. The tetracyclic skeleton forms strong π-π interactions with Trp167, and the 11- and 12-nitrogen atoms of **6a** form hydrogen bonds with the side chain of Glu473 through a water molecule. One of the carbonyl oxygen atoms in the tetracyclic backbone also forms a hydrogen bond with T276 through water-mediated network, while another carbonyl oxygen atom forms a direct hydrogen bond with the backbone of Trp275. The pyridine moiety inserts into a pocket at the bottom of the binding cleft, which is formed by Tyr390, Met388, and Trp539. The pyridine ring forms π-π stacking and π-S interactions with Trp539 and Met388, respectively. In addition, the nitrogen atom of the pyridine also forms a hydrogen bond with Glu315 through a water-mediated network. Since the *Sm*ChiA catalytic cleft is acidic, the nitrogen atom of pyridine moiety may guide the inhibitor to bind in the substrate-binding site.

As we failed to obtain the crystal structure of *Of*Chi-h in complex with **6a**, molecular docking was used to investigate its binding mode. The docking results revealed that **6a** binds to at the −3, −2 and −1 subsites of *Of*Chi-h in a manner similar to *Sm*ChiA. The 3-pyridine group inserts into a binding cleft formed by Tyr156, Phe184, and Met381. The nitrogen atom forms a hydrogen bond with the key catalytic residue Glu308, as observed with inhibitor **2–8-s2** (Figures 4 and 5(a)). This interaction is likely responsible for the higher inhibitory activity against *Of*Chi-h and explains the unsuccessful attempts to replace the 3-pyridine group. The tetracyclic skeleton of **6a** forms strong π-π stacking interaction with Trp160, while a second carbonyl oxygen forms a hydrogen bond with backbone of the Trp268. In contrast, molecular docking showed that **6e** binds to *Of*Chi-h in a different manner (Figure 5(b)). The tetracyclic skeleton of **6e** is sandwiched between Trp268 and Trp389, forming strong π-π stacking interactions similar to that of **2–8-s2.** The trimethoxybenzene group inserts into −2 subsites of *Of*Chi-h, because it is too bulky to fit into the binding cleft formed by Tyr156, Phe184, and Met381.

## Conclusion

In summary, this study demonstrates the successful ­development of conformationally restricted dipyridopyrimidine-3-carboxamide derivatives as potent inhibitors targeting *Of*Chi-h. By employing a conformational restriction strategy, we discovered novel *Of*Chi-h inhibitors with significantly improved inhibitory activity. The lead compound **6a** exhibited exceptional potency (*K_i_* = 58 nM) against *Of*Chi-h, establishing its potential as a sustainable pesticide candidate. Notably, **6a** also demonstrated promising activity against human chitinase *Hs*Chit1 (*K_i_* = 295 nM), suggesting that this scaffold could also be repurposed to target human chitinases implicated in inflammatory diseases^10^^,^^32–34^. Structural investigations through molecular docking and high-resolution co-crystallization with *Sm*ChiA revealed that **6a** binds to *Of*Chi-h via a novel mechanism, occupying subsites −3 to −1 of the substrate-binding cleft. This binding mode not only validates the design rationale but also highlights the advantage of rigidifying the inhibitor scaffold to enhance target engagement. The fused tetracyclic skeleton introduced here serves as a robust structural template for designing selective chitinase inhibitors. Overall, this work underscores the broader utility of conformational restriction as a strategic tool in chitinase inhibitor discovery, bridging rational design with mechanistic validation, and paves the way for broader adoption of conformationally constrained scaffolds in chitinase inhibitors design.

## Experimental section

### Instruments and chemicals

Unless otherwise noted, standard and analytical graded reagents and solvents were obtained from Bide Pharmatech Ltd (Shanghai, China) and used without further purification. Ethanol, dichloromethane, methanol, and dimethyl sulfoxide were purchased from Shanghai Titan Scientific Co., Ltd. (Shanghai, China), TLC plates (Silica gel 60 F254) and chromatography silica gel (200–300 mesh) were supplied by Qingdao Haiyang Chemical (Qingdao, China), *N,N*-Dimethylformamide dimethyl acetal (DMFDMA), were sourced from Aladdin Biochemical Technology Co., Ltd. (Shanghai, China). All reactions were under ambient air. Melting points (M.p.) were recorded on Büchi B540 apparatus (Büchi Labortechnik, Flawil, Switzerland) and are uncorrected. High-resolution electron mass spectra (HRMS) were performed on Waters, Xevo G2 TOF spectrometer (MA, USA).^1^H NMR,^19^F NMR and ^13^C NMR spectra were recorded on Bruker AM-400 and Bruker ASCEND 600 MHz (^1^H at 400 MHz, ^13^C at 100 MHz or 150 MHz, ^19^F at 376 MHz) spectrometers with DMSO-*d*_6_ or CDCl_3_ as solvents and TMS as internal standard. Chemical shifts are reported in *δ* (parts per million). The following abbreviations were used to explain the multiplicities: s = singlet, d = doublet, t = triplet, q = quartette, m = multiplet, coupling constant (Hz) and integration.

### General synthetic procedure of target compounds 6a-6l

Detailed synthesis procedures of compounds **5a-5l** were described in our previous report^11^. Subsequently, to a round-bottom flask were added compound 5 (0.5 mmol, 1.0 equiv) and 1,1-dimethoxy-*N,N*-dimethylmethanamine (DMFDMA, 2.0 ml, approx. 15 mmol, ∼30 equiv). The mixture was stirred under neat conditions at 110 °C for 10–60 min (monitored by TLC). After completion, the reaction mixture was cooled to room temperature, and ethanol (2 ml) was added. The resulting solid was collected by filtration, washed with cold ethanol (2 × 1 ml), and dried under vacuum. The crude product was further purified by column chromatography on silica gel using DCM/MeOH (30:1) as eluent to afford the final compounds **6a–6l** as yellow solids.

### Analysis data of compounds 6a-6l

**6a**, Yellow solid, Yield: 67.3%, M.p.: 352.1–352.7 °C; ^1^H NMR (400 MHz, DMSO-*d*_6_) δ 9.56 (t, *J* = 5.8 Hz, 1H), 8.93 (s, 1H), 8.74 (d, *J* = 7.1 Hz, 1H), 8.64 (s, 1H), 8.53 (d, *J* = 3.8 Hz, 1H), 7.88 (d, *J* = 7.9 Hz, 1H), 7.78 (d, *J* = 6.7 Hz, 1H), 7.48 (dd, *J* = 7.8, 4.9 Hz, 1H), 7.08 (t, *J* = 7.0 Hz, 1H), 4.52 (d, *J* = 5.7 Hz, 2H), 2.47 (s, 3H). ^13^C NMR (100 MHz, DMSO) δ 174.45, 166.09, 160.98, 157.66, 150.70, 147.89, 147.19, 143.29, 138.33, 136.58, 136.42, 135.25, 133.18, 125.47, 123.96, 113.42, 110.48, 100.85, 40.33, 17.82. HRMS-ESI (m/z): calcd for C_20_H_15_N_6_O_2_ [M + H]^+^, 371.1256; found, 371.1257.

**6b**, Yellow solid, Yield: 41.7%, M.p.: 302.5–304.7 °C; ^1^H NMR (400 MHz, Chloroform-*d*) δ 9.79 (s, 1H), 8.82 (d, *J* = 7.1 Hz, 1H), 8.74 (s, 1H), 8.63 (d, *J* = 4.0 Hz, 1H), 8.35 (s, 1H), 7.74 (d, *J* = 8.1 Hz, 1H), 7.63 (d, *J* = 6.7 Hz, 1H), 7.37 (dd, *J* = 7.9, 4.9 Hz, 1H), 6.97 (t, *J* = 7.0 Hz, 1H), 6.31 (q, *J* = 7.1 Hz, 1H), 2.70 (s, 3H), 1.95 (d, *J* = 7.2 Hz, 3H). ^13^C NMR (150 MHz, CDCl_3_) δ 161.48, 160.31, 159.65, 159.35, 151.84, 150.23, 150.01, 148.83, 142.07, 136.21, 135.60, 134.81, 134.45, 125.25, 123.84, 114.69, 113.58, 110.96, 50.79, 29.65, 18.84. HRMS-ESI (m/z): calcd for C_21_H_17_N_6_O_2_ [M + H]^+^, 385.1413; found, 385.1412.

**6c**, Yellow solid, Yield: 53.4%. M.p.: 306.1–307.0 °C; ^1^H NMR (400 MHz, DMSO-*d*_6_) δ 9.25 (s, 1H), 8.87 (d, *J* = 1.6 Hz, 2H), 8.71 (m, 1H), 8.62 − 8.56 (m, 2H), 7.82 (m, 1H), 7.11 (t, *J* = 7.0 Hz, 1H), 5.44 (s, 2H), 2.53 (s, 3H).^13^C NMR (100 MHz, DMSO) *δ* = 161.62, 160.10, 159.17, 158.87, 154.60, 151.13, 150.90, 144.12, 144.01, 143.84, 139.36, 136.86, 133.75, 125.34, 114.14, 113.72, 110.52, 48.35, 18.05. HRMS-ESI (m/z): calcd forC_19_H_14_N_7_O_2_ [M + H]^+^, 372.1209; found, 372.1210;

**6d**, Yellow solid, Yield: 72.4%, M.p.: 303.8–304.5 °C; ^1^H NMR (400 MHz, Chloroform-*d*) δ 9.78 (s, 1H), 8.82 (d, *J* = 7.2 Hz, 1H), 8.45 (s, 1H), 7.62 (d, *J* = 6.7 Hz, 1H), 7.47 − 7.30 (m, 5H), 6.96 (t, *J* = 7.0 Hz, 1H), 5.22 (s, 2H), 2.70 (s, 3H). ^13^C NMR (100 MHz, CDCl_3_) δ 160.69, 159.44, 152.56, 141.97, 139.56, 136.15, 135.64, 134.93, 129.90, 129.26, 129.12, 128.76, 128.38, 125.30, 115.18, 113.57, 110.91, 49.85, 29.34. HRMS-ESI (m/z): calcd for C_21_H_16_N_5_O_2_ [M + H]^+^, 370.1304; found, 370.1305;

**6e**, Yellow solid, Yield: 54.8%, M.p.: 329.8–331.1 °C; ^1^H NMR (400 MHz, Chloroform-*d*) δ 9.79 (s, 1H), 8.82 (d, *J* = 7.0 Hz, 1H), 8.45 (s, 1H), 7.62 (d, *J* = 6.7 Hz, 1H), 6.96 (t, *J* = 7.0 Hz, 1H), 6.62 (s, 2H), 5.13 (s, 2H), 3.86 (s, 6H), 3.83 (s, 3H).^13^C NMR (150 MHz, CDCl_3_) δ 161.97, 160.62, 159.54, 159.36, 153.76, 152.37, 151.81, 141.96, 138.30, 136.22, 135.56, 130.35, 125.24, 115.04, 113.59, 110.86, 105.56, 60.83, 56.21, 50.04, 18.82. HRMS-ESI (m/z): calcd for C_24_H_22_N_5_O_5_[M + H]^+^, 460.1621; found, 460.1620.

**6f**, Yellow solid, Yield: 59.1%, M.p.: 303.9–305.1 °C; ^1^H NMR (400 MHz, Chloroform-*d*) δ 9.78 (s, 1H), 8.81 (d, *J* = 7.1 Hz, 1H), 8.44 (s, 1H), 7.01 − 6.91 (m, 3H), 6.86 (d, *J* = 8.1 Hz, 1H), 5.14 (s, 2H), 3.88 (s, 3H), 3.88 (s, 3H), 2.69 (s, 3H). ^13^C NMR (100 MHz, CDCl_3_) δ 162.12, 160.73, 159.59, 159.44, 152.47, 151.83, 149.56, 149.49, 141.91, 136.12, 135.64, 127.34, 125.28, 121.14, 115.17, 113.55, 111.63, 111.44, 110.88, 56.02, 55.98, 49.76, 18.85. HRMS-ESI (m/z): calcd for C_23_H_20_N_5_O_4_[M + H]^+^, 430.1515; found, 430.1514.

**6g**, Yellow solid, Yield: 68.3%, M.p.: 311.8–314.6 °C; ^1^H NMR (400 MHz, Chloroform-*d*) δ 9.77 (s, 1H), 8.81 (d, *J* = 7.1 Hz, 1H), 8.44 (s, 1H), 7.61 (d, *J* = 6.6 Hz, 1H), 7.36 (d, *J* = 8.6 Hz, 2H), 6.92 (m,6.87–6.98 3H), 5.15 (s, 2H), 3.80 (s, 3H), 2.69 (s, 3H). ^13^C NMR (100 MHz, CDCl_3_) δ 173.25, 160.43, 157.25, 154.87, 153.95, 149.83, 148.66, 147.36, 135.67, 135.01, 134.18, 133.97, 125.34, 123.44, 113.80, 100.67, 93.58, 38.62, 29.42, 17.89. HRMS-EI (m/z): calcd for C_22_H_17_N_5_O_3_[M^] +^, 399.1331; found, 399.1335.

**6h**, Yellow solid, Yield: 42.1%. M.p.: 324.6–326.3 °C; ^1^H NMR (400 MHz, Chloroform-*d*) δ 9.78 (s, 1H), 8.82 (d, *J* = 6.8 Hz, 1H), 8.42 (s, 1H), 7.62 (d, *J* = 6.8 Hz, 1H), 7.19 − 7.09 (m, 2H), 6.96 (t, *J* = 7.0 Hz, 1H), 6.76 (d, *J* = 7.6 Hz, 1H), 6.71 − 6.59 (m, 3H), 5.11 (s, 2H), 2.70 (s, 3H). ^13^C NMR (150 MHz, CDCl_3_) δ 160.64, 159.52, 159.38, 152.61, 151.76, 147.17, 141.89, 136.07, 136.01, 135.56, 130.15, 129.83, 125.23, 118.19, 115.25, 115.13, 114.50, 113.49, 110.81, 49.64, 29.65. HRMS-ESI (m/z): calcd for C_21_H_17_N_6_O_2_[M + H]^+^, 385.1413; found, 385.1412.

**6i**, Yellow solid, Yield: 46.2%. M.p.: 353.5–354.5 °C; ^1^H NMR (400 MHz, Chloroform-*d*) δ 9.77 (s, 1H), 8.82 (d, *J* = 7.2 Hz, 1H), 8.52 (s, 1H), 7.63 (d, *J* = 6.7 Hz, 1H), 7.31 − 7.27 (m, 2H), 7.20 − 7.13 (m, 1H), 6.97 (t, *J* = 7.0 Hz, 1H), 5.34 (s, 2H), 2.70 (s, 3H). ^19^F NMR (376 MHz, DMSO) δ −115.22. ^13^C NMR (150 MHz, CDCl_3_) δ 161.93, 160.63, 159.62, 159.33, 158.47 (d, *J* = 250.0 Hz), 152.50, 151.84, 141.93, 136.23, 135.61, 134.51, 129.85 (d, *J* = 5.2 Hz), 128.13 (d, *J* = 8.0 Hz), 125.94 (d, *J* = 3.6 Hz),125.24, 116.86 (d, *J* = 21.3 Hz), 114.95, 113.60, 110.89, 47.21, 29.65. HRMS-ESI (m/z): calcd for C_21_H_14_^35^ClFN_5_O_2_ [M + H]^+^, 422.0820; found, 422.0821; calcd for C_21_H_14_^37^ClFN_5_O_2_ [M + H]^+^, 424.0791; found, 424.0795.

**6j**, Yellow solid, Yield: 43.4%. M.p.: 323.4–324.6 °C; ^1^H NMR (400 MHz, DMSO-*d*_6_) δ 10.89 (s, 1H), 9.38 (s, 1H), 8.80 − 8.71 (m, 1H), 8.40 (s, 1H), 7.84 (dt, *J* = 6.7, 1.4 Hz, 1H), 7.60 (d, *J* = 7.9 Hz, 1H), 7.35 (dt, *J* = 8.1, 1.1 Hz, 1H), 7.18 (d, *J* = 2.4 Hz, 1H), 7.13 (t, *J* = 7.0 Hz, 1H), 7.07 (ddd, *J* = 8.1, 6.9, 1.2 Hz, 1H), 6.95 (ddd, *J* = 7.9, 7.0, 1.0 Hz, 1H), 4.27 (t, *J* = 7.3 Hz, 2H), 3.17 (t, *J* = 7.3 Hz, 2H), 2.54 (s, 3H). ^13^C NMR (150 MHz, DMSO) δ 166.56, 163.44, 160.71, 160.24, 157.97, 153.85, 150.96, 146.18, 139.79, 136.64, 130.06, 129.37, 127.37, 123.88, 122.21, 121.90, 121.49, 118.83, 118.54, 110.40, 110.36, 53.14, 29.38, 18.24. HRMS-ESI (m/z): calcd for C_24_H_19_N_6_O_2_[M + H]^+^, 423.1569; found, 423.1571.

**6k**, Yellow solid, Yield: 33.4%. M.p.: 297.3–298.5 °C; ^1^H NMR (400 MHz, Chloroform-*d*) δ 9.76 (s, 1H), 8.81 (d, *J* = 6.9 Hz, 1H), 8.50 (s, 1H), 7.62 (d, *J* = 6.7 Hz, 1H), 7.47 − 7.37 (m,1H), 6.96 (t, *J* = 7.0 Hz, 1H), 6.53 (d, *J* = 3.1 Hz, 1H), 6.42 − 6.33 (m, 1H), 5.21 (s, 2H), 2.70 (s, 3H). ^13^C NMR (150 MHz, CDCl_3_) δ 161.99, 160.21, 159.51, 159.34, 152.26, 151.79, 147.51, 143.56, 141.86, 136.15, 135.56, 125.23, 115.04, 113.54, 110.87, 110.80, 110.59, 42.13, 18.80. HRMS-ESI (m/z): calcd for C_19_H_14_N_5_O_3_[M + H]^+^, 360.1097; found, 360.1098.

**6l**, Yellow solid, Yield: 34.2%. M.p.: 200.6.-201.3 °C; ^1^H NMR (400 MHz, Chloroform-*d*) δ 9.78 (s, 1H), 8.83 (d, *J* = 7.3 Hz, 2H), 8.67 (s, 1H), 7.64 (d, *J* = 6.8 Hz, 2H), 6.97 (t, *J* = 6.8 Hz, 2H), 4.84 (d, *J* = 2.4 Hz, 3H), 2.71 (s, 3H), 2.57 (s, 1H). ^13^C NMR (150 MHz, CDCl_3_) δ 161.94, 159.93, 159.63, 159.35, 151.86, 151.25, 141.89, 136.25, 135.61, 129.87, 125.26, 114.64, 113.62, 76.09, 75.55, 35.38, 18.80. HRMS-ESI (m/z): calcd for C_17_H_12_N_5_O_2_ [M + H]^+^, 318.0991; found, 318.0995.

Full ^1^H, ^13^C, ^19^F NMR and HRMS raw spectra of compound **6a-6l** are included in (Supplementary Material Figures S7–S43).

### Preparation and purification of enzyme

*Sm*ChiA was expressed in *Escherichia coli* BL21(DE3), *Hs*Chit1 and *Of*Chi-h were overexpressed in *Pichia pastoris* GS115. The *Escherichia coli* BL21(DE3) strain was purchased from TaKaRa Biotech (Dalian, 6dChina) while the *Pichia pastoris* GS115 strain was obtained from Invitrogen (Thermo Fisher Scientific, CA, USA), both of which were cultured and handled according to the protocols provided by suppliers. All chitinases were purified using immobilised metal ion affinity chromatography as previously described^35^. Briefly, solid ammonium sulphate was slowly added to the culture supernatant to achieve 75% saturation. After incubation at 4 °C for 24 h, the precipitated proteins were collected by centrifugation at 12,000 × g for 30 min at 4 °C. The pellet was resuspended thoroughly in buffer A (20 mM sodium phosphate, 0.5 M sodium chloride, pH 7.4), and the solution was centrifuged again at 12,000 × g for 15 min at 4 °C to remove insoluble material. The clarified supernatant was loaded onto a 5 ml HisTrap^™^ Crude affinity column (GE Healthcare) that had been pre-equilibrated with buffer A. The column was subsequently washed with buffer A containing 75 mM imidazole to remove non-specifically bound proteins. Recombinant chitinases were eluted using buffer A containing 250 mM imidazole. The expressed proteins were quantified using a BCA protein assay kit (TaKaRa Biotech, China). And their purities were determined by SDS-PAGE and found to be >95% in all cases.

### Chitinase inhibition assay and K_i_ determination

Chitinase activities for *Sm*ChiA, *Hs*Chit1 and *Of*Chi-h were determined using 4-methylumbelliferyl-*N, N′*-diacetyl-*β*-D-chitobioside (MU-*β*-(GlcNAc)_2_) (purchased from Sigma) as a substrate. The final reaction mixture volume used for inhibitor screening was 100 μL, consisting of 10 nM enzyme, 4 μM MU-β-(GlcNAc)_2_, 10 μM inhibitor, and 2% DMSO in a 20 mM sodium phosphate buffer (pH 6.0). The reaction in the absence of inhibitor was used as a positive control. After incubation at 30 °C for 20 min, 0.5 M sodium carbonate was added to the reaction mixture, and the fluorescence produced by the released 4-MU was quantified with a Varioskan Flash microplate reader (Thermo Fisher Scientific) using excitation and emission wavelengths of 360 and 450 nm, respectively. Experiments were performed in triplicate unless specified otherwise. For *K_i_* value determination, the inhibitor concentration was varied in the above reaction, and different concentrations of substrate (2–8 μM) were used. The amount of released 4-MU was quantified as above, and the *K_i_* value and the mode of inhibition were determined using the Dixon plot.

### Molecular docking

Protein structure for molecular docking was prepared using the protein preparation wizard in Maestro 10.2 (2015–2 release). The crystal structure of *Of*Chi-h in complex with inhibitor **2–8-s2** (PDB: 6jmn) was used in molecular docking study. Bond orders were assigned, hydrogens were added and waters beyond 5 Å from inhibitor **2–8-s2** were deleted. The protein structure was the refined using PROPKA and minimised OPLS3^36^ force field. The docking grid were generated by Receptor Grid Generation tool integrated in Glide.

The grid box dimensions were determined based on the coordinates of the bound **2–8-s2.** A 10 Å*10 Å*10 Å inner grid box and a 20 Å*20 Å*20 Å outer grid box was used without other constraints. The 2D structures of **6a-6l** were constructed by Chemdraw 17.1 and were imported to Maestro 10.2. Initial ligand conformations are minimised and sampled by MacroModel, which were then prepared LigPrep tool. Hydrogen atoms were added while the ionisation and tautomeric states of ligands at a target pH of 7.0 ± 2.0 were generated using Epik. The 3D geometry of each structure was optimised by OPLS-3 force field. Glide in extra precision (XP) mode was used in docking. Molecular mechanics-generalized Born surface area (MM-GBSA) method in Prime was used for rescoring the docked pose of ligand. For each compound, the pose with the lowest score was retained.

### Cocrystallization and data collection

Protein-inhibitor complex crystal was prepared by co-crystallization methods. Briefly, the crystal of *Sm*ChiA-6a crystal complex was obtained in a solution consisting of 5 mM **6a**, 5% (v/v) DMSO, 0.75 M sodium citrate (pH 7.2) and 20% (v/v) methanol by the vapour method^37^. The crystals were soaked for 10 s in a reservoir solution containing 20% (v/v) glycerol as a cryoprotection reagent, and subsequently flash-cooled in liquid nitrogen. X-ray diffraction data of the complexes were collected on the BL-19U1 at Shanghai Synchrotron Radiation Facility in China. The diffraction data of the complexes were processed using the HKL-3000 package.

### Structure determination and refinement

The structures of *Sm*ChiA-**6a** was solved by molecular replacement with Phaser^38^ using the structure of free *Sm*ChiA as a model (PDB entry 2WLZ). The PHENIX suite of programs^39^ was used for structure refinement. Coot was used for manually building and extending the molecular models^40^. The stereo chemical quality of the models was checked by PROCHECK^41^. The coordinates of *Sm*ChiA-6a were deposited in the Protein Data Bank as entry 7FD6. The structural figures were generated using the PyMOL program. The statistics for the diffraction data and the structure refinement are summarised in Table 2.

**Table 2.** X-ray data collection and structure-refinement statistics of the ­complex ***Sm*ChiA-6a**.

	*Sm*ChiA-6a
Protein Data Bank entry	7FD6
Space group	C222_1_
Unit-cell parameters	
a (Å)	132.525
b (Å)	202.186
c (Å)	59.43
α (°)	90
β (°)	90
γ (°)	90
Wavelength (Å)	0.97776
Temperature (K)	100
Resolution (Å)	34.92–1.786 (1.85–1.786)
Unique reflections	74412
Observed reflections	117961
*R*_merge_	0.03271 (0.01386)
Average multiplicity	1.6 (1.0)
<σ(I)>	14.76 (3.08)
Completeness (%)	98 (82)
*R/R*_free_	0.1748/ 0.1917
Protein atoms	4124
Water molecules	678
Other atoms	28
R.M.S. deviation from ideal	
Bond lengths (Å)	0.004
Bond angles (°)	0.682
Wilson B factor (Å^2^)	18.57
Average B factor (Å^2^)	23.32
Protein atoms	21.64
Water molecules	33.20
Ligand molecules	31.57
Ramachandran plot (%)	
Favored	99
Allowed	1.5
Outliers	0

## Supplementary Material

Supplementary Material anonymous.docx

## Data Availability

The data that support the findings of this study are available from the corresponding author upon reasonable request.
