# Supplementary material for: Discovery of conformation constrained tetracyclic compounds as potent chitinase OfChi-h inhibitors with a novel binding mode
Source: J Enzyme Inhib Med Chem. 2025 Jul 7;40(1):2528056. doi: 10.1080/14756366.2025.2528056 (PMC12239106; doi:10.1080/14756366.2025.2528056)
Supplement: Supplementary Material anonymous.docx [file IENZ_A_2528056_SM0067.docx]

**Supplementary Information**

**Discovery of conformation constrained tetracyclic compounds as potent chitinase *Of*Chi-h inhibitors with a novel binding mode**

**Table of Contents**

[1 Dixon plots for inhibition chitinase *Of*Chi-h by **6a-6l** 3](#_Toc193907752)

[2 Materials and Methods 5](#_Toc193907753)

[3 ^1^H, ^13^C, ^19^F NMR and HRMS Spectra 6](#_Toc193907754)

# Dixon plots for inhibition chitinase *Of*Chi-h by 6a-6l

Figure S 1 Dixon plots for inhibition chitinase *Of*Chi-h by 6a and 6b. The trend lines represent three substrate concentrations.

Figure S 2 Dixon plots for inhibition chitinase *Of*Chi-h by 6c and 6d. The trend lines represent three substrate concentrations.

Figure S 3 Dixon plots for inhibition chitinase *Of*Chi-h by 6e and 6f. The trend lines represent three substrate concentrations.

Figure S 4 Dixon plots for inhibition chitinase *Of*Chi-h by 6g and 6h. The trend lines represent three substrate concentrations.

Figure S 5 Dixon plots for inhibition chitinase *Of*Chi-h by 6i and 6j. The trend lines represent three substrate concentrations.

Figure S 6 Dixon plots for inhibition chitinase *Of*Chi-h by 6k and 6l. The trend lines represent three substrate concentrations.

# Materials and Methods

Unless otherwise noted, standard and analytical graded reagents and solvents were obtained from commercial sources and used without further purification. All reactions were under air atmosphere. ^1^H NMR, ^19^F NMR and ^13^C NMR spectra were recorded on Bruker AM-400 (^1^H at 400 MHz, ^13^C at 100 MHz, ^19^F at 376 MHz) spectrometer with DMSO-*d*_6_ or CDCl_3_ as solvent and TMS as internal standard. Chemical shifts are reported in *δ* (parts per million). High-resolution electron mass spectra (HRMS) was performed on Waters, Xevo G2 TOF spectrometer (MA, USA). Analytical thin-layer chromatography (TLC) was performed on precoated plates (silica gel 60 F254), and spots were visualized with ultraviolet (UV) light. The following abbreviations were used to explain the multiplicities: s = singlet, d = doublet, t = triplet, q = quartet, m = multiplet, coupling constant (Hz) and integration.

# ^1^H, ^13^C, ^19^F NMR and HRMS Spectra


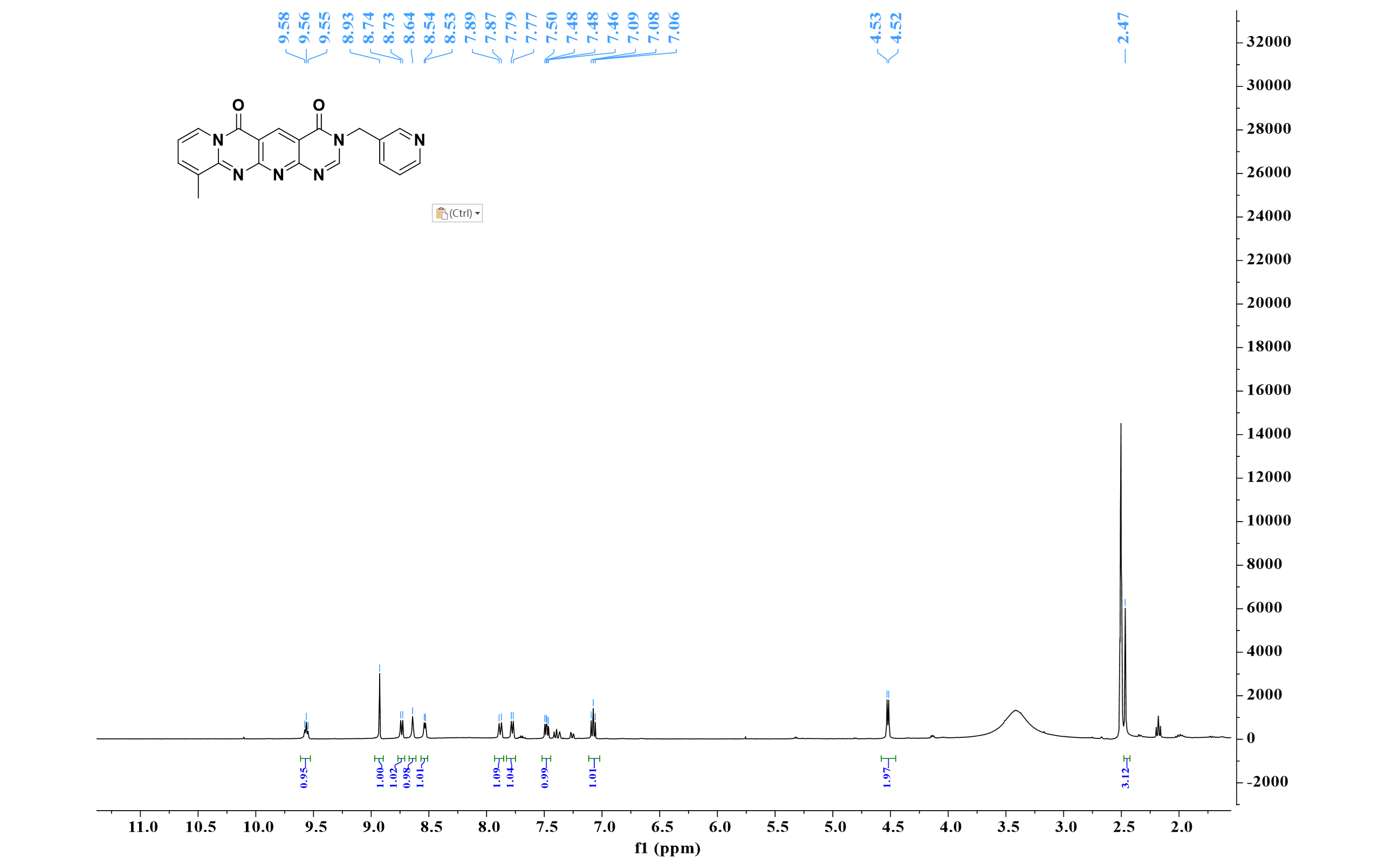


Figure S 7 ^1^H NMR (400 MHz, DMSO-*d*_6_) spectrum of compound 6a


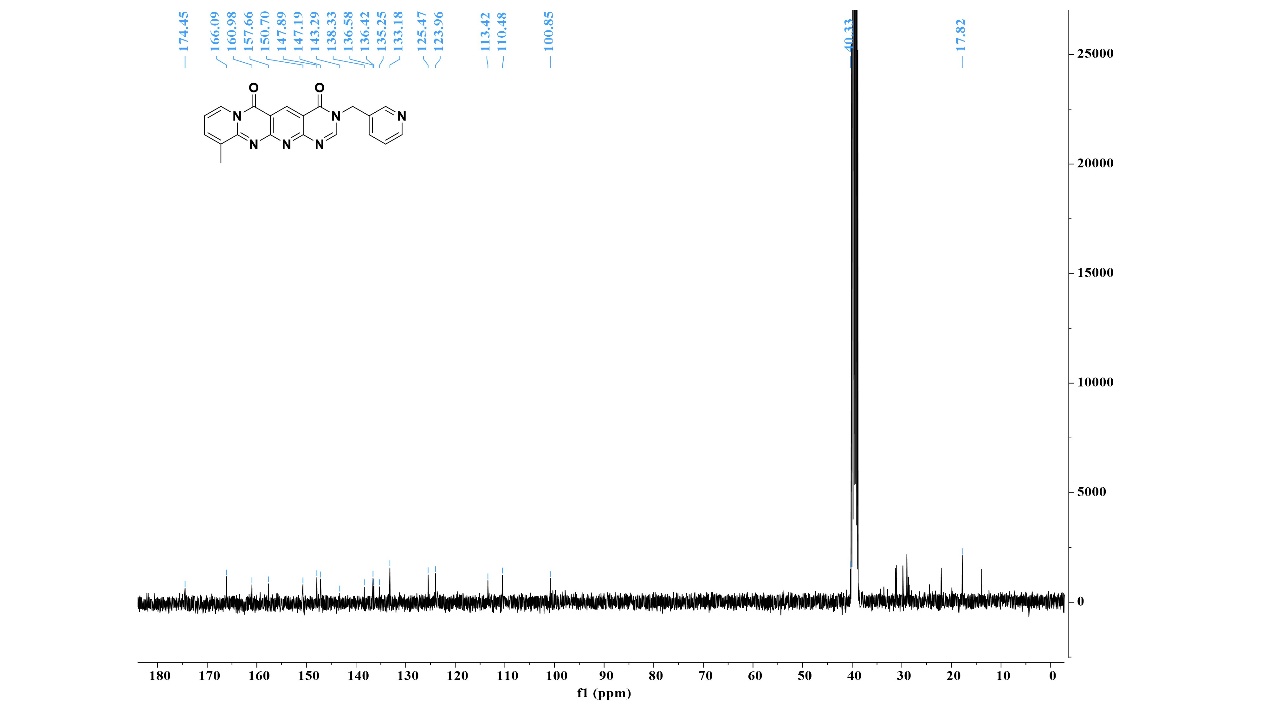


Figure S 8 ^13^C NMR (150 MHz, DMSO) spectrum of compound 6a


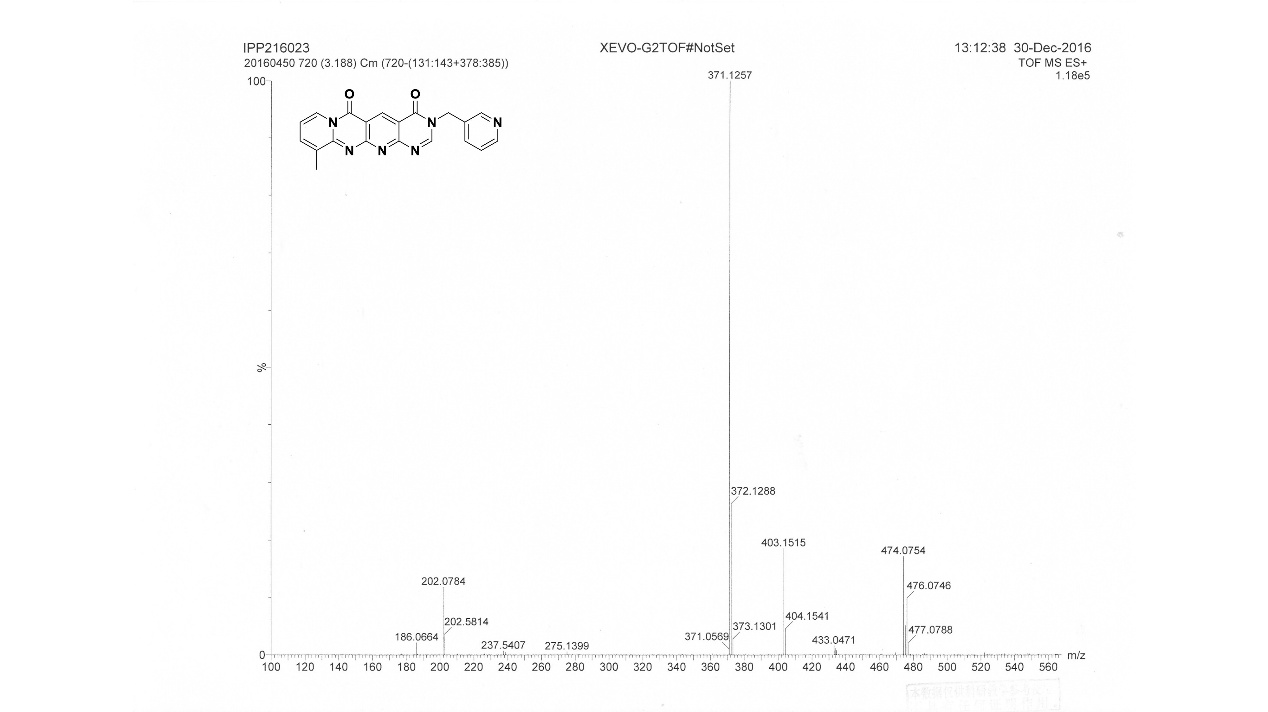


Figure S 9 HRMS of compound 6a


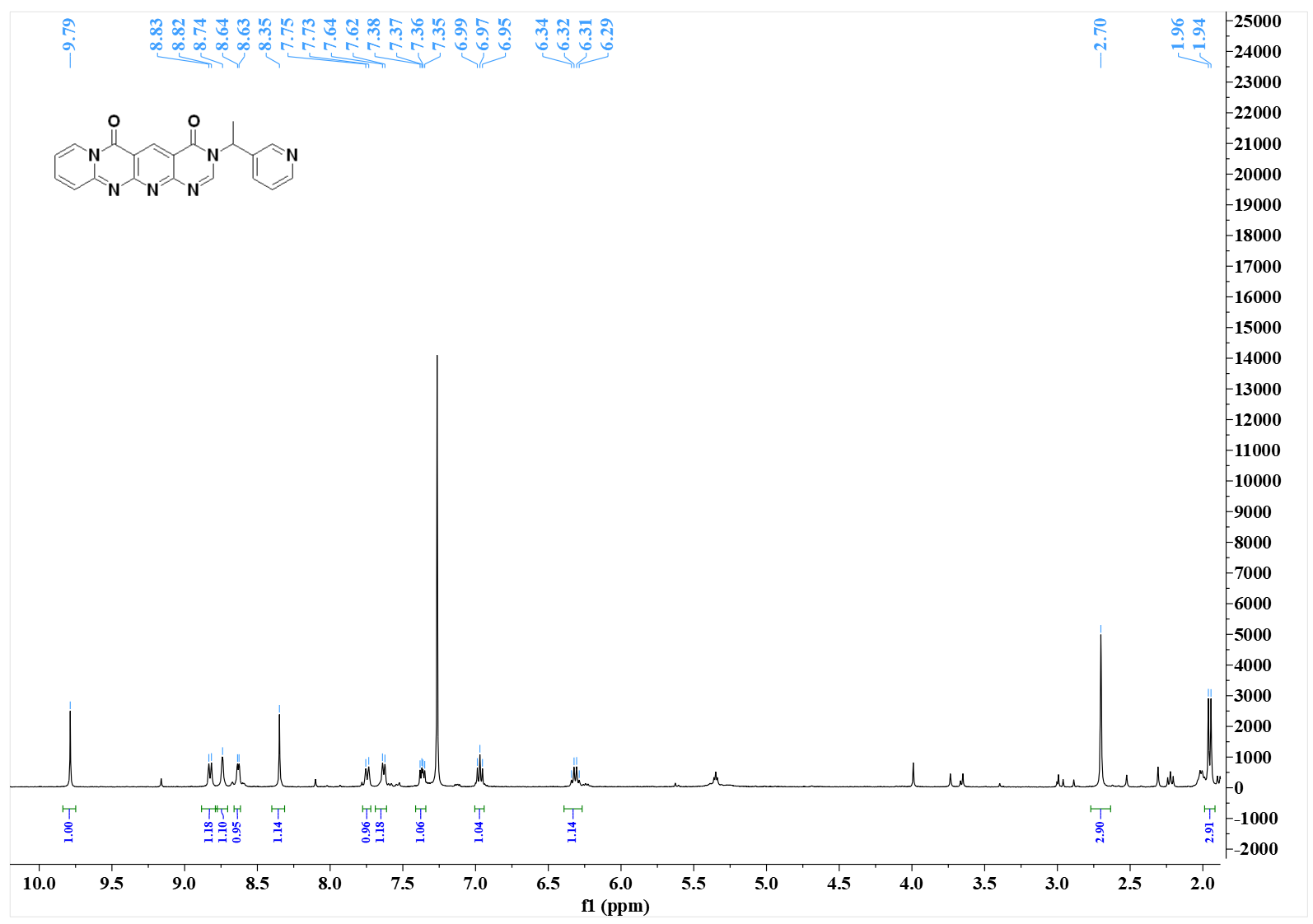


Figure S 10 ^1^H NMR (400 MHz, CDCl_3_) spectrum of compound 6b


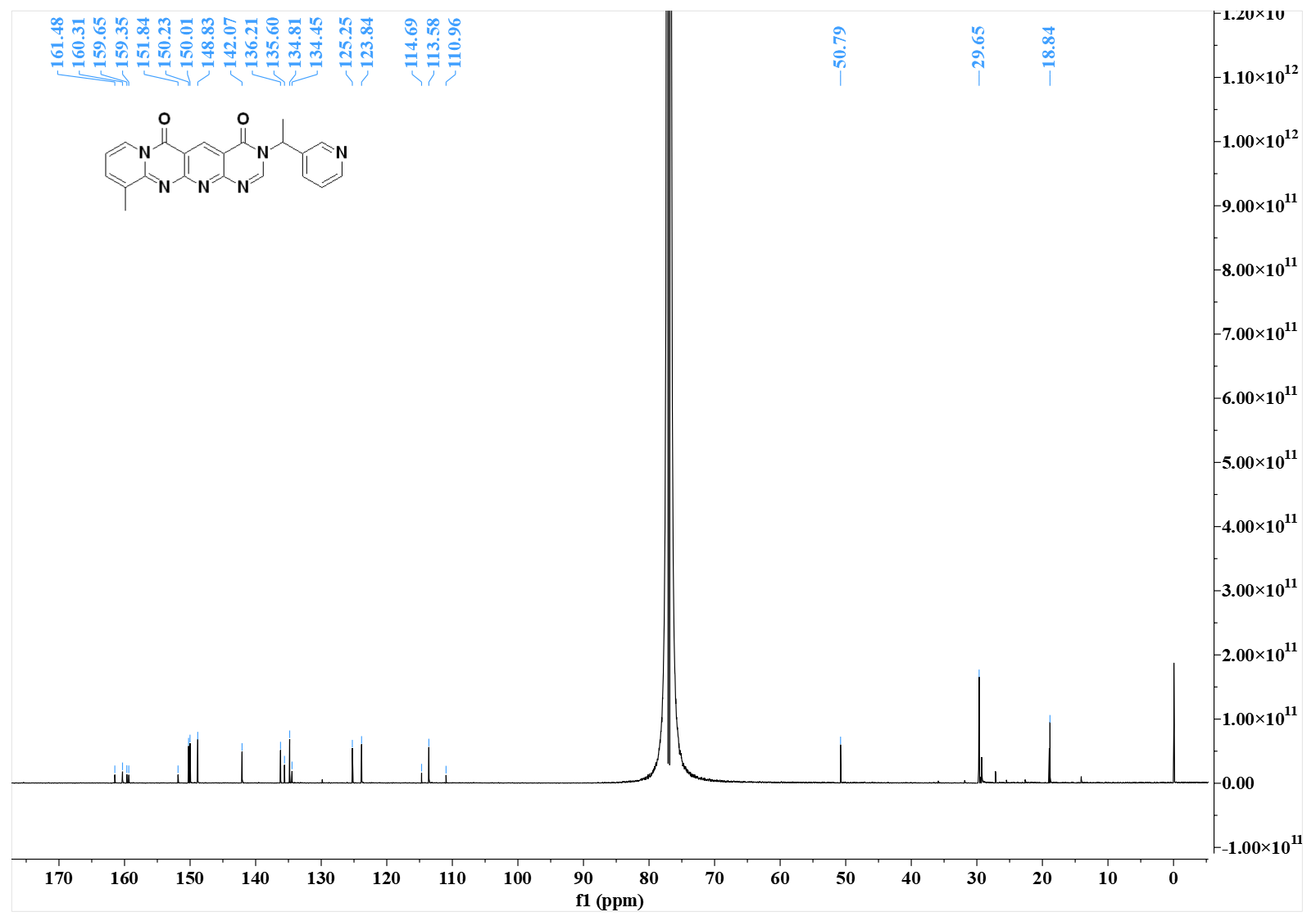


Figure S 11 ^13^C NMR (150 MHz, CDCl_3_) spectrum of compound 6b


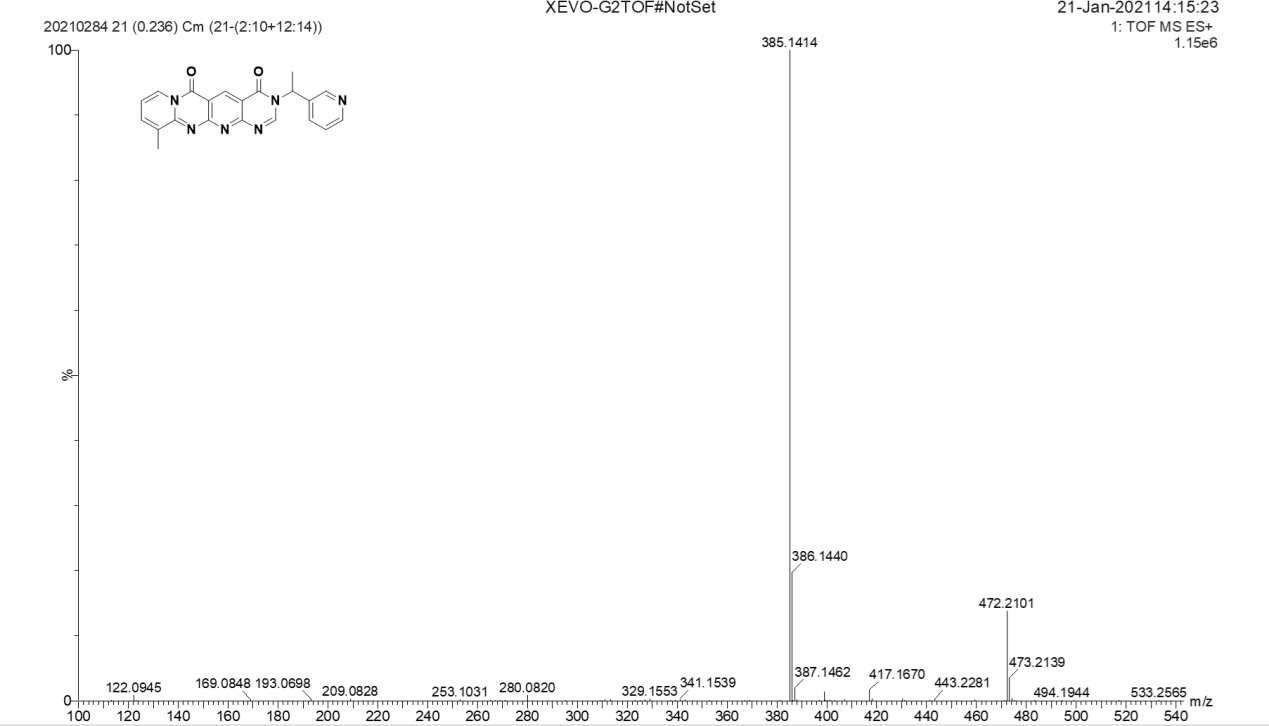


Figure S12 HRMS of compound 6b


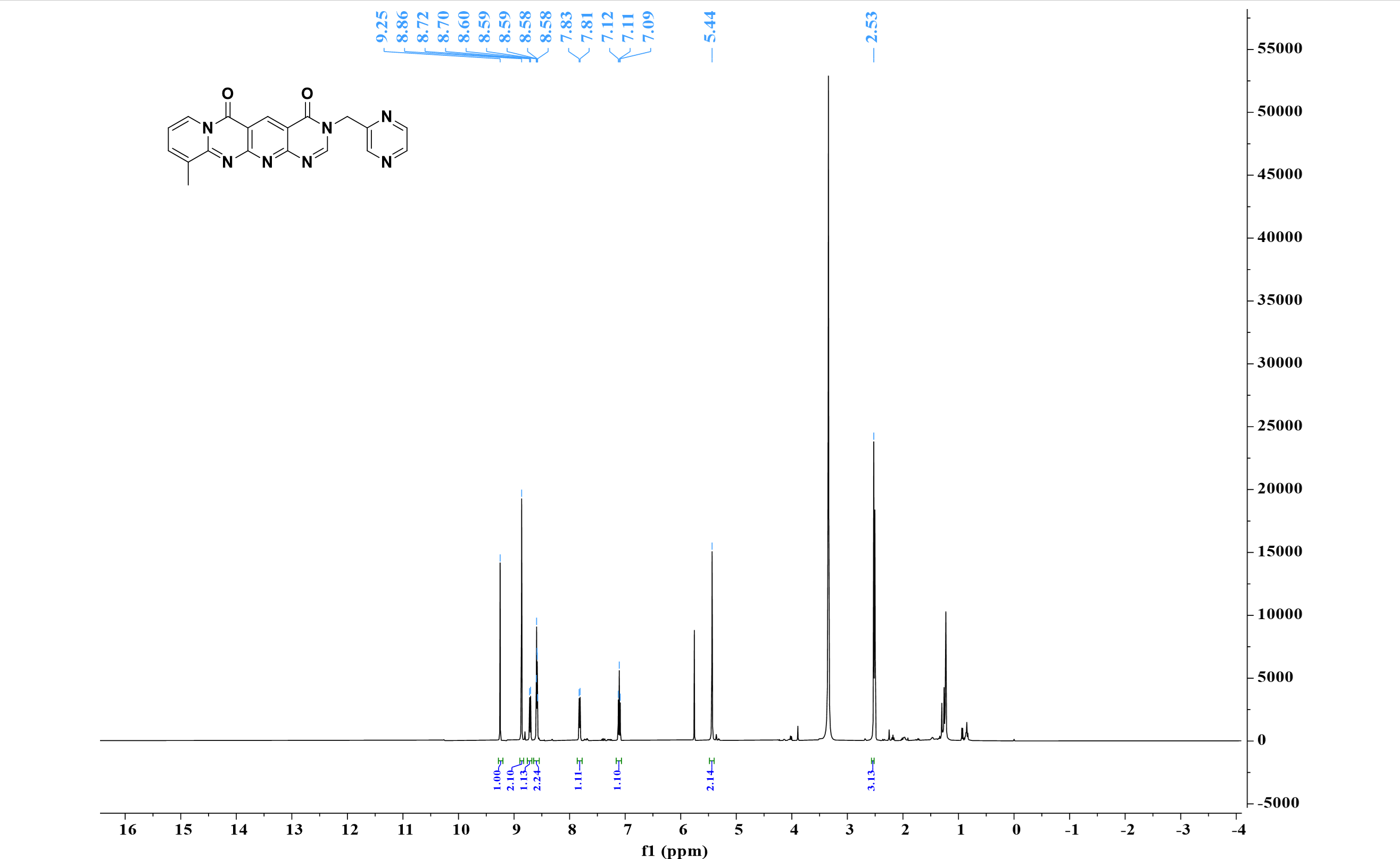


Figure S 13 ^1^H NMR (400 MHz, DMSO-*d*_6_) spectrum of compound 6c


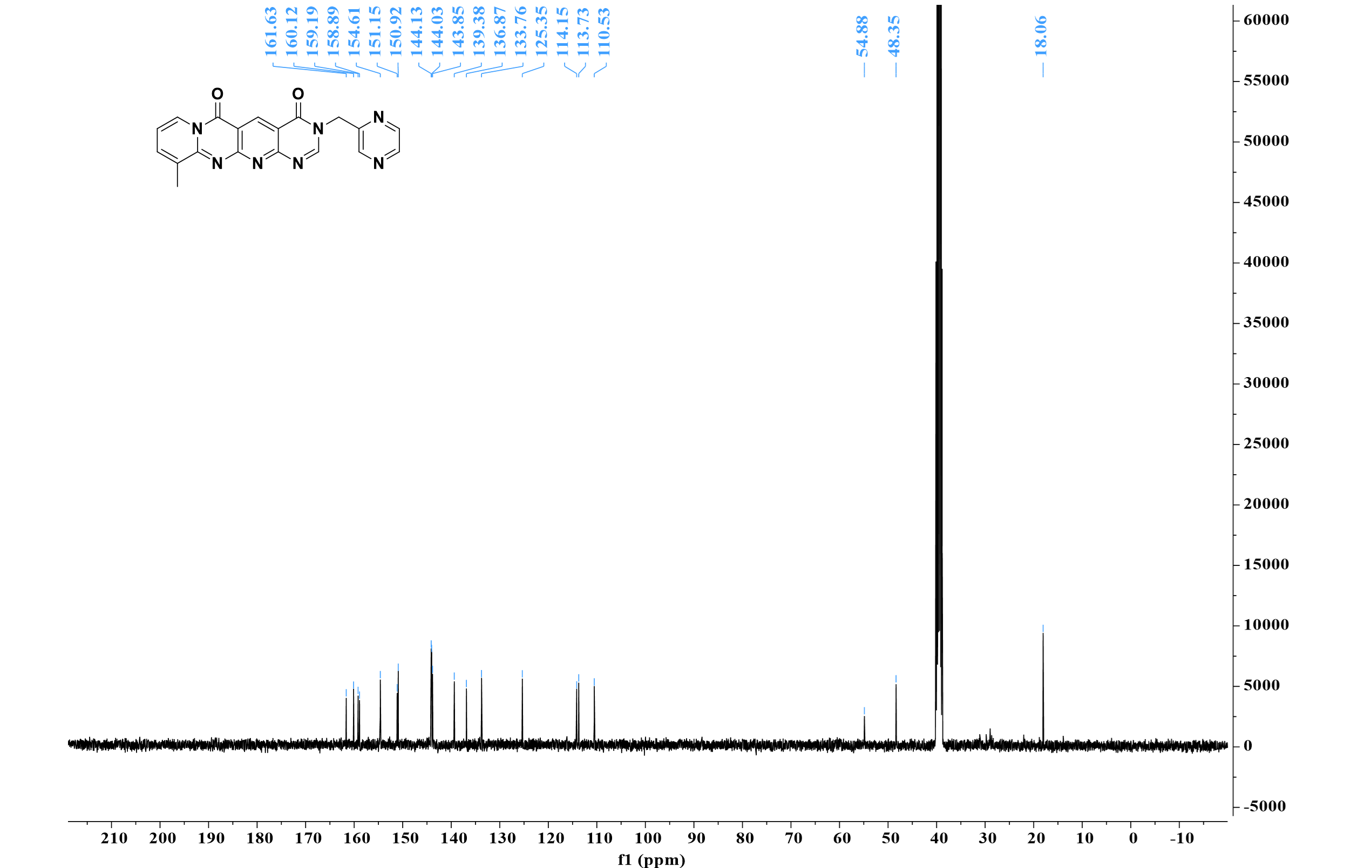


Figure S 14 ^13^C NMR (100 MHz, DMSO) spectrum of compound 6c


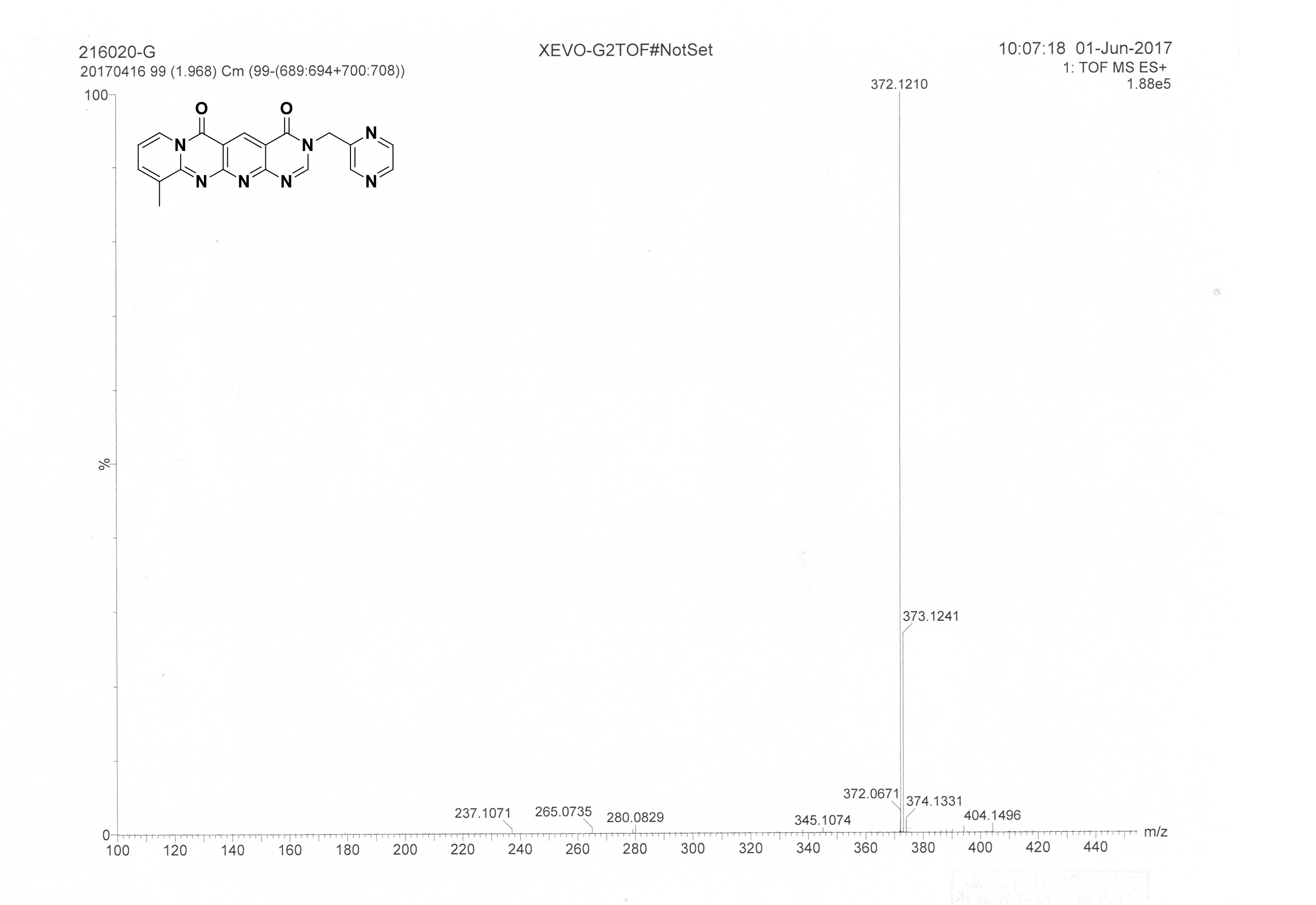


Figure S 15 HRMS of compound 6c


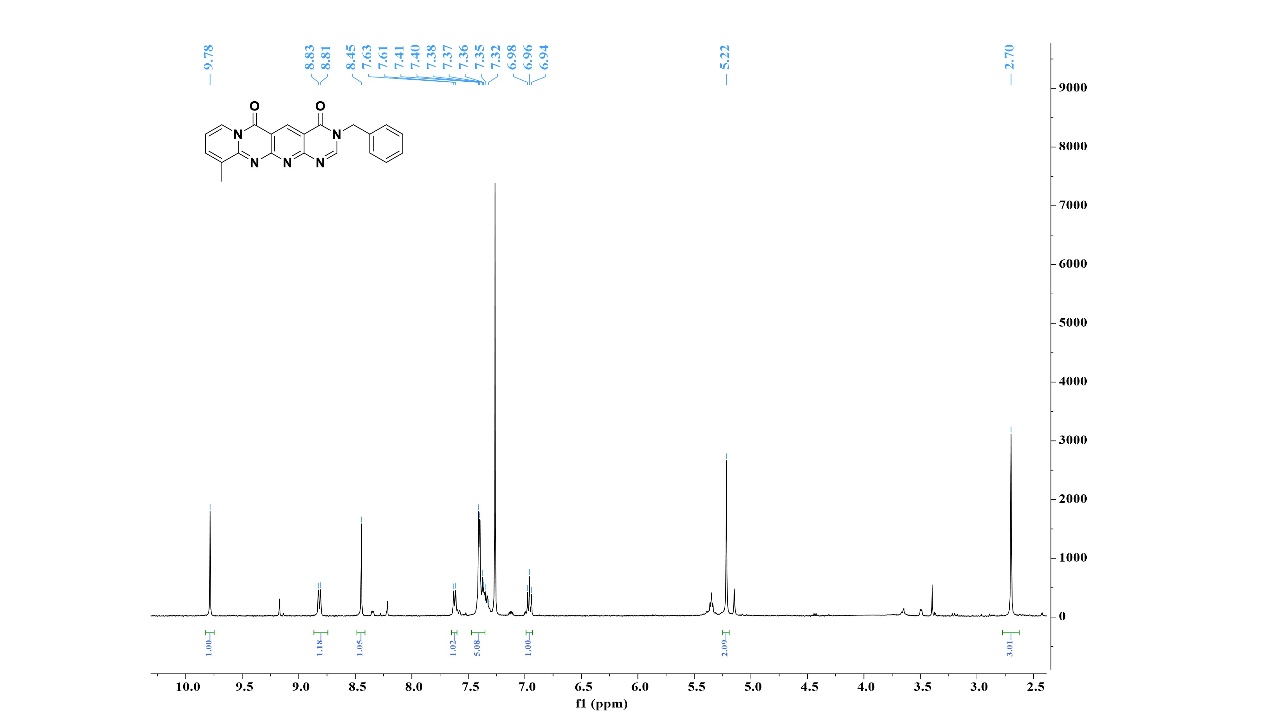


Figure S 16 ^1^H NMR (400 MHz, CDCl_3_) spectrum of compound 6d


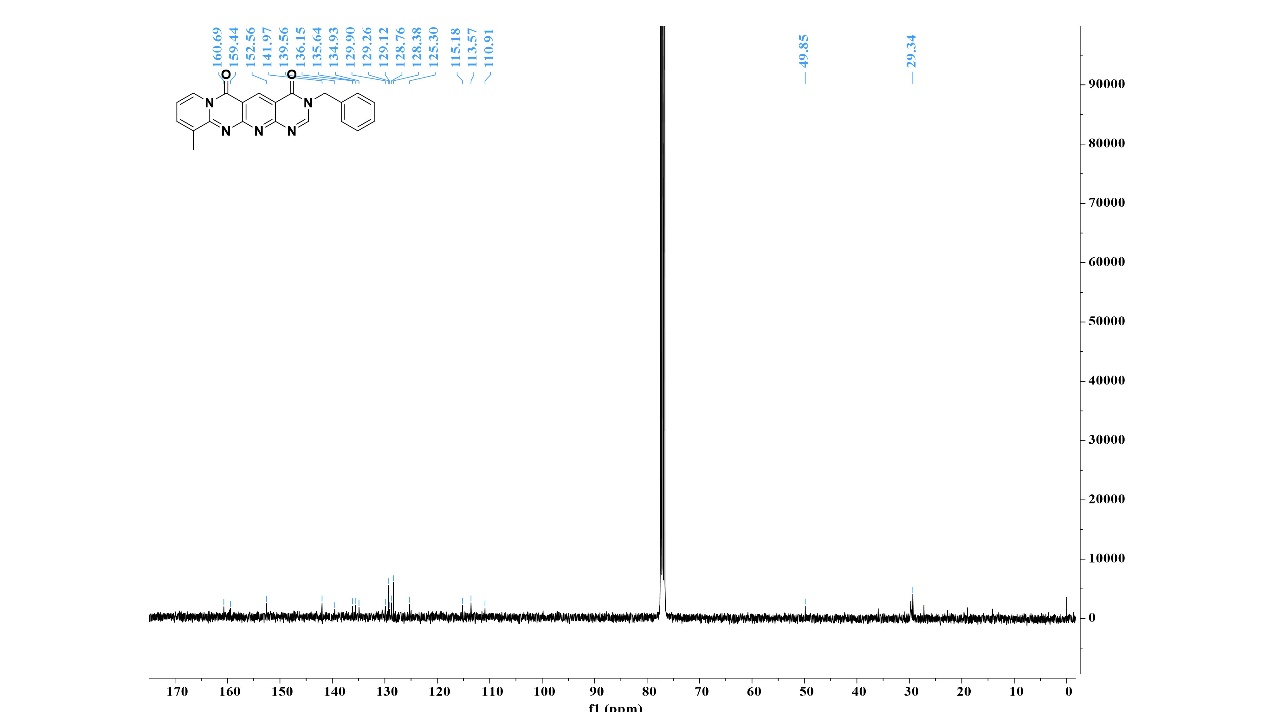


Figure S 17 ^13^C NMR (100 MHz, CDCl_3_) spectrum of compound 6d


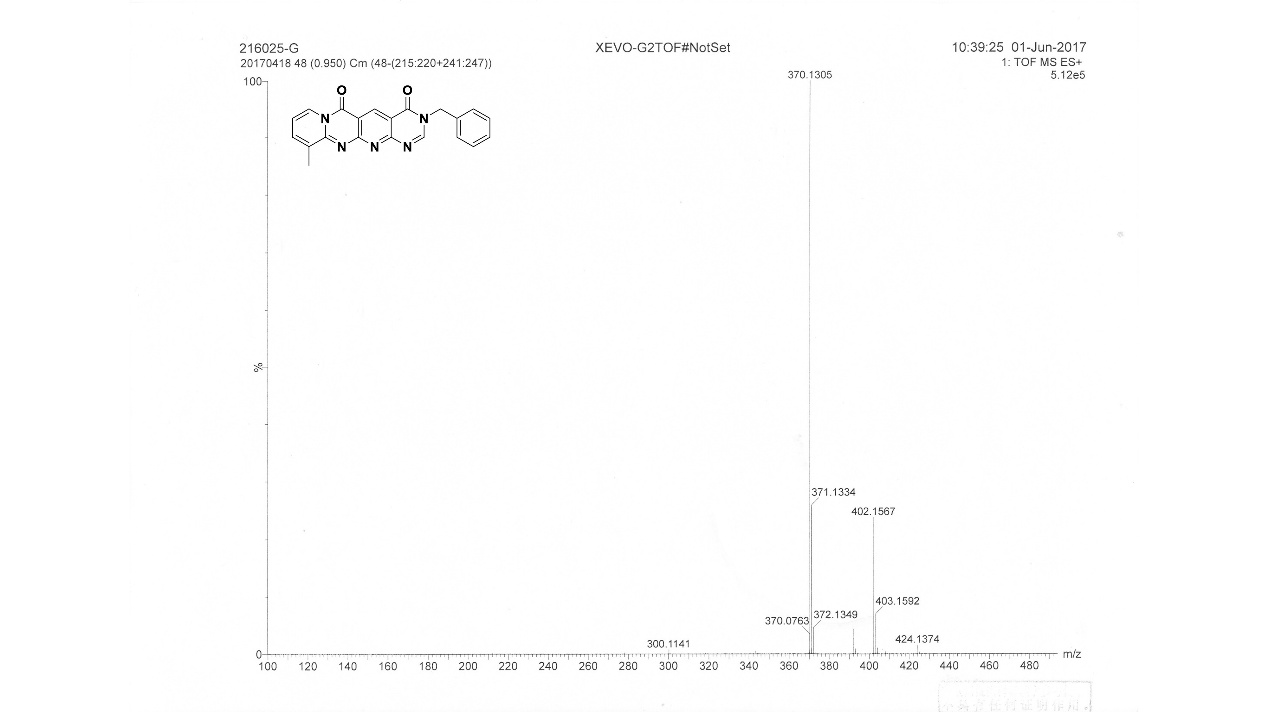


Figure S 18 HRMS of compound 6d


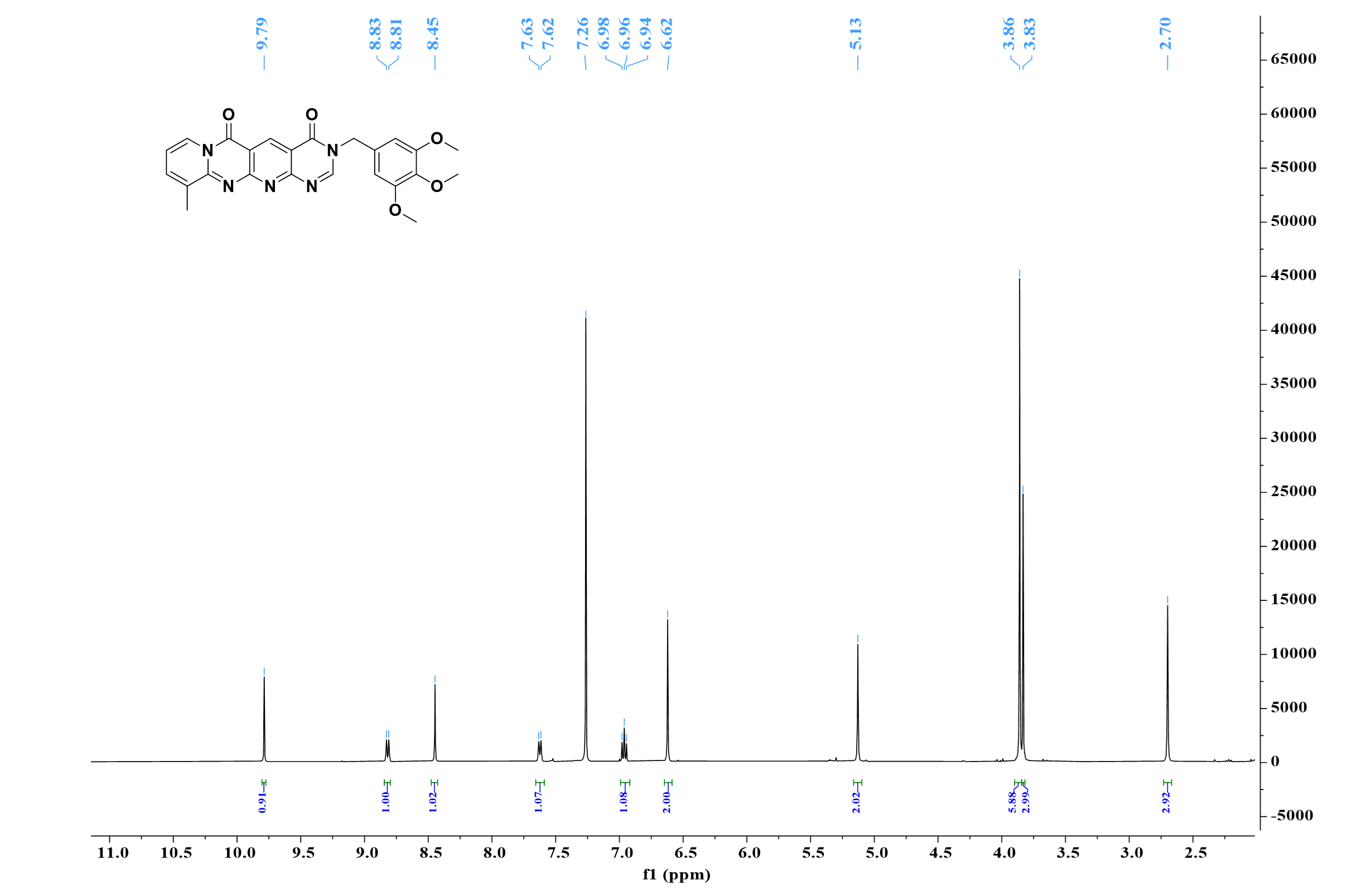


Figure S 19 ^1^H NMR (400 MHz, CDCl_3_) spectrum of compound 6e


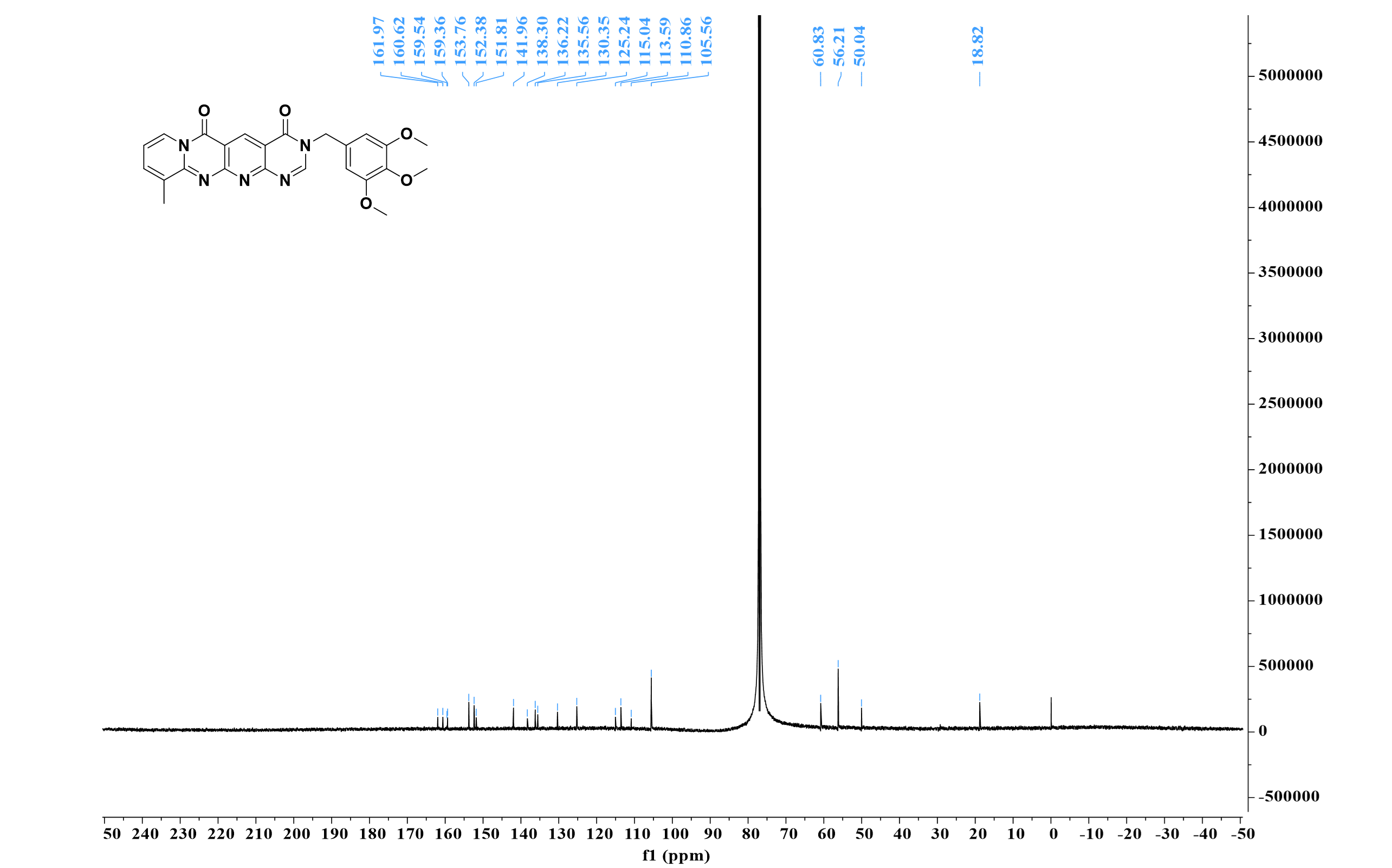


Figure S 20 ^13^C NMR (150 MHz, CDCl_3_) spectrum of compound 6e


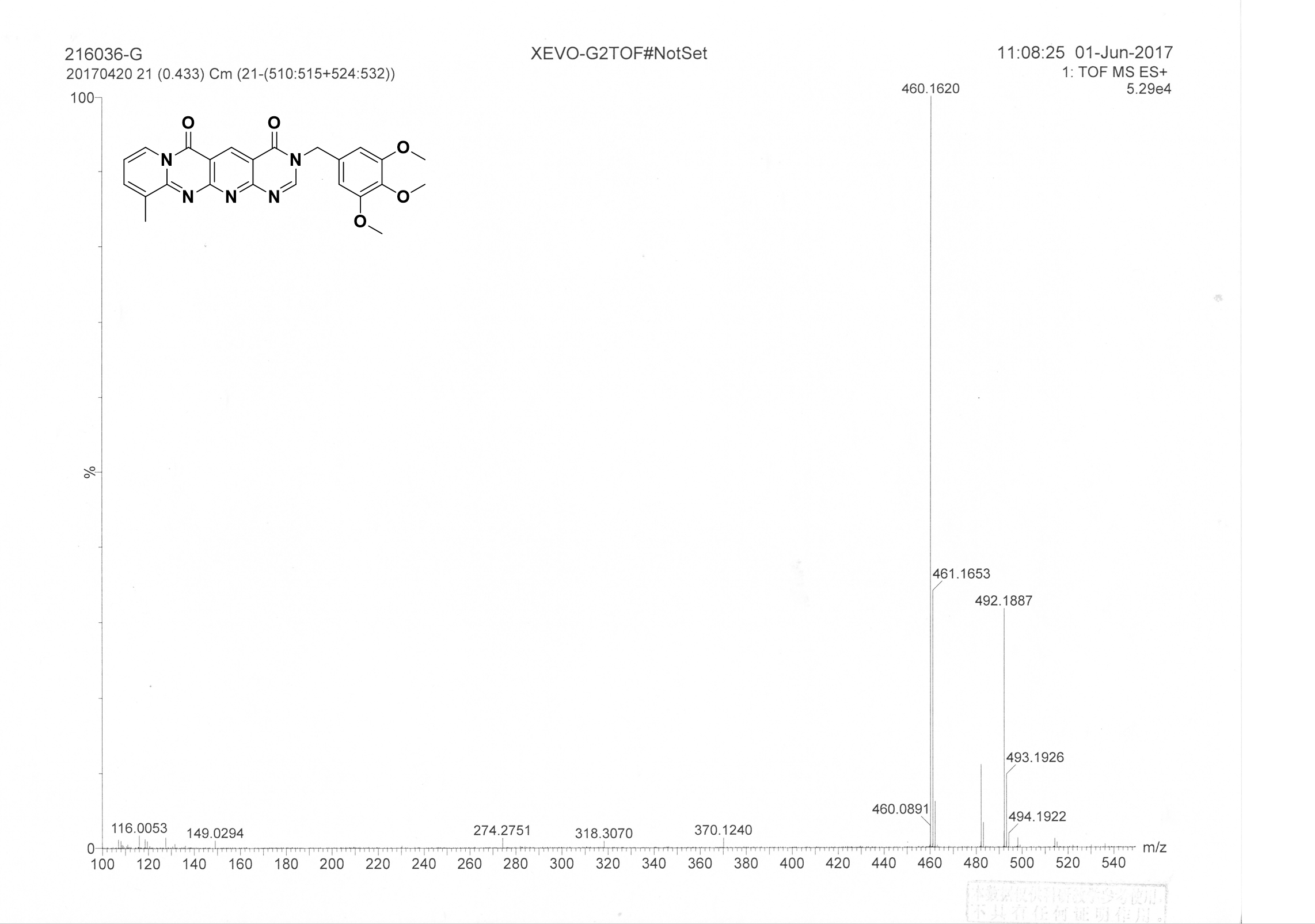


Figure S 21 HRMS of compound 6e


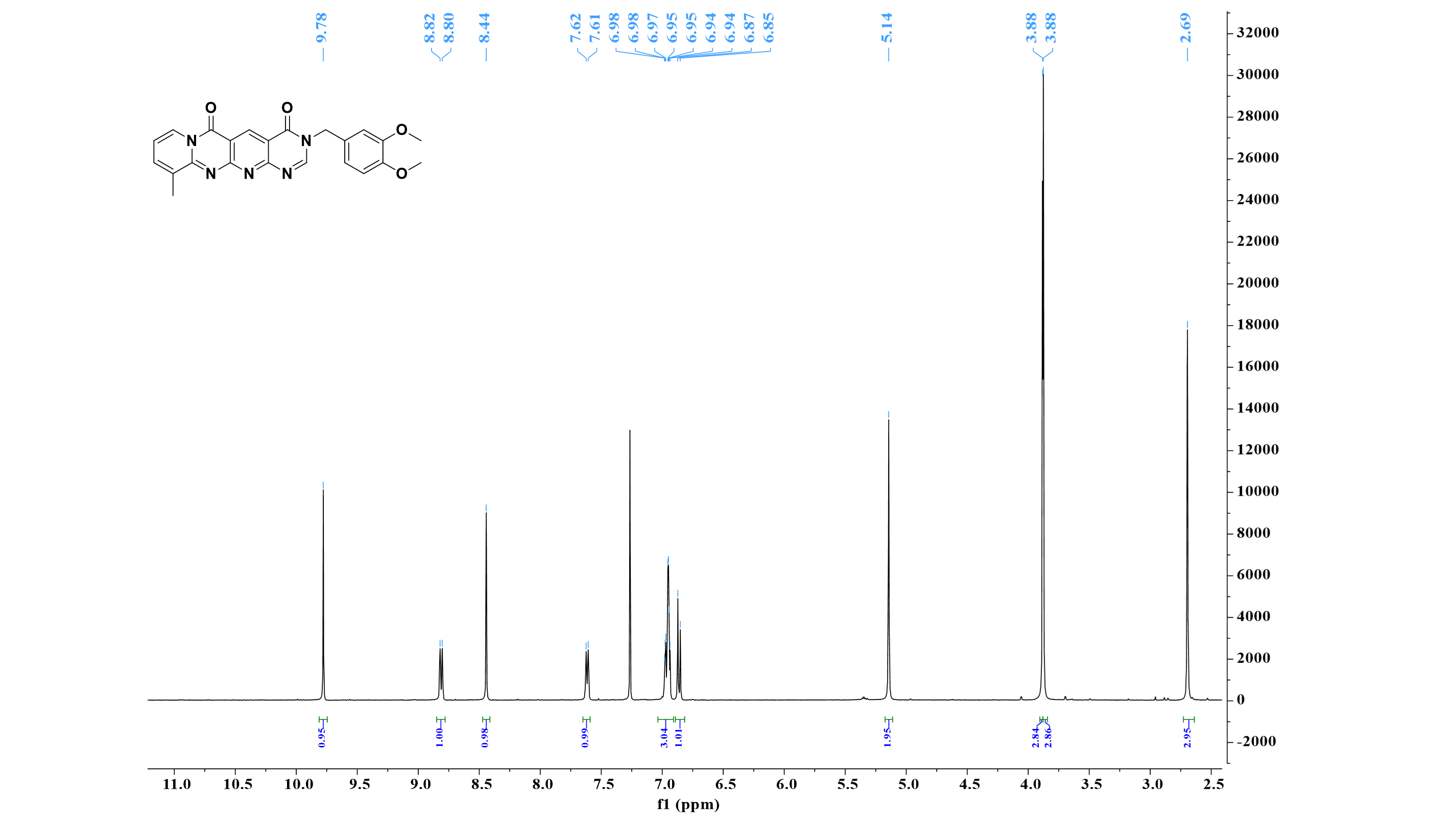


Figure S 22 ^1^H NMR (400 MHz, CDCl_3_) spectrum of compound 6f


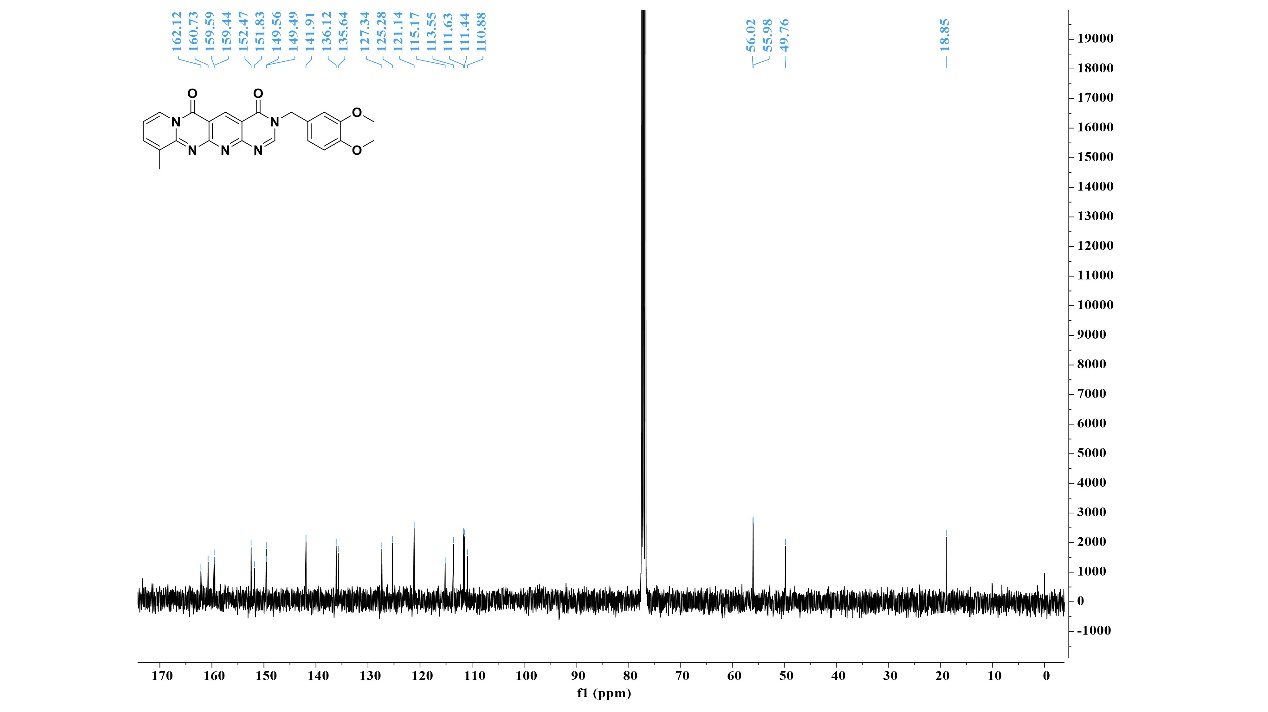


Figure S 23 ^13^C NMR (100 MHz, CDCl_3_) spectrum of compound 6f


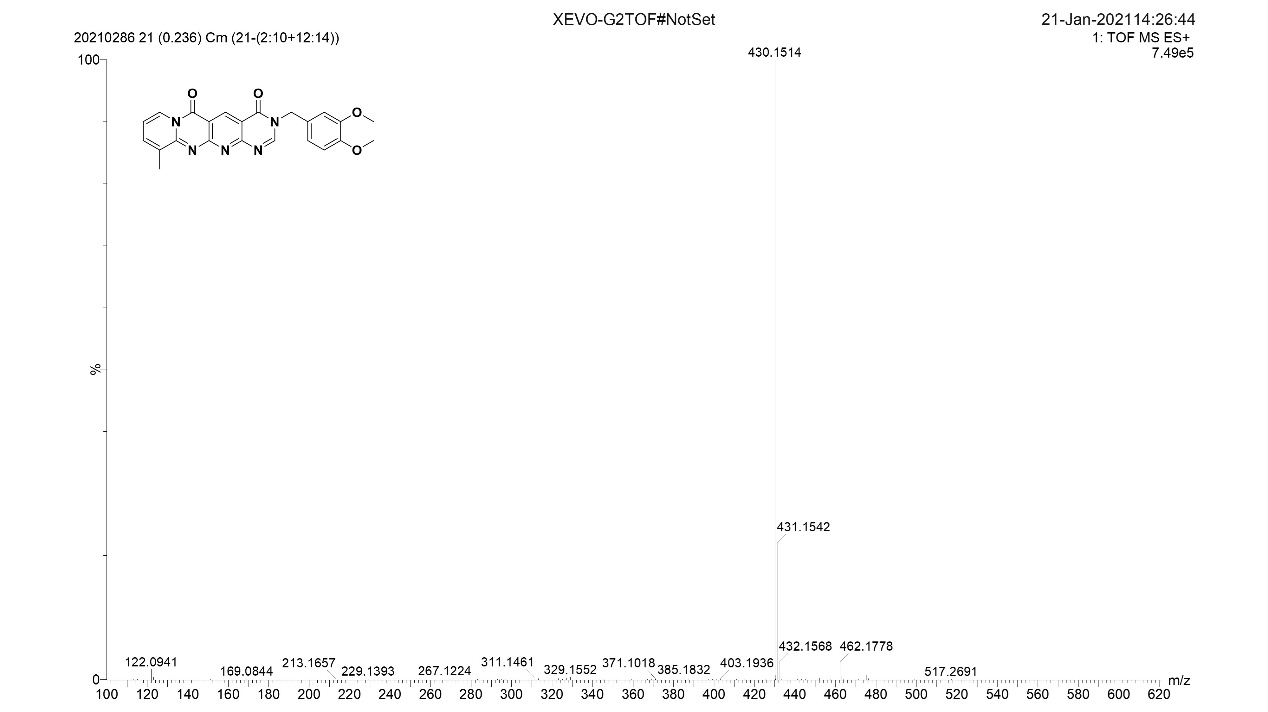


Figure S 24 HRMS of compound 6f


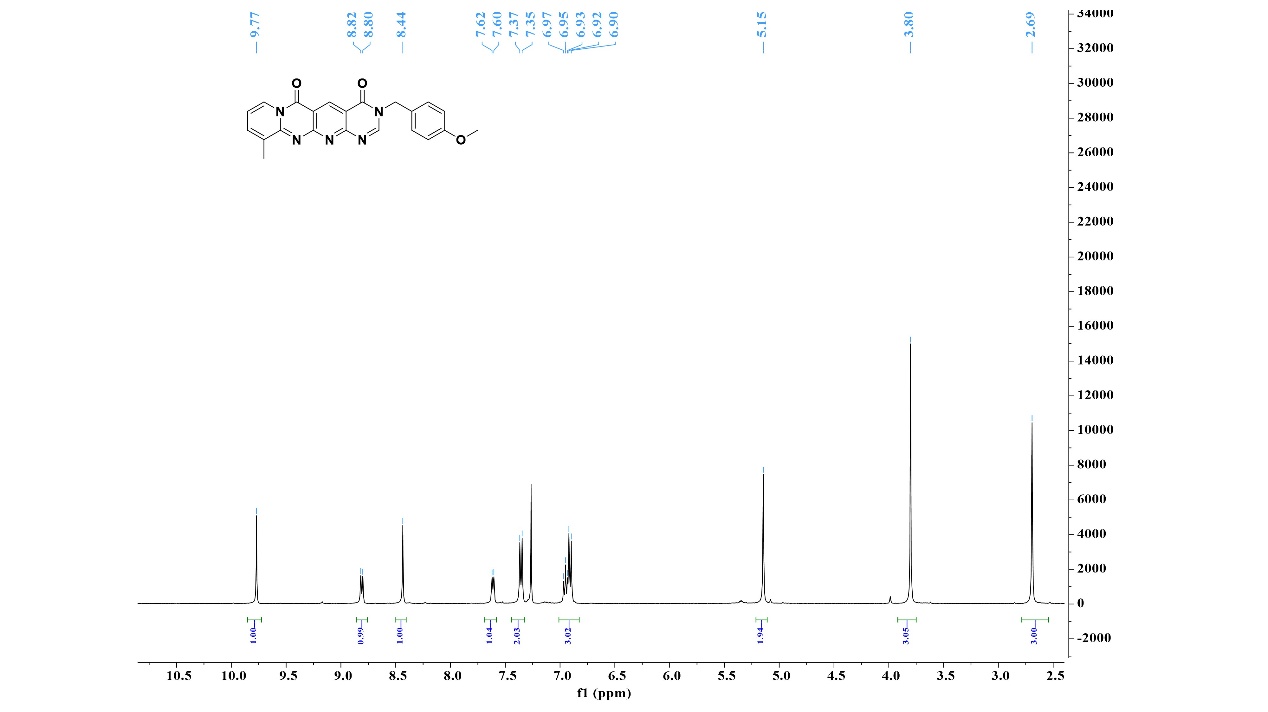


Figure S 25 ^1^H NMR (400 MHz, CDCl_3_) spectrum of compound 6g


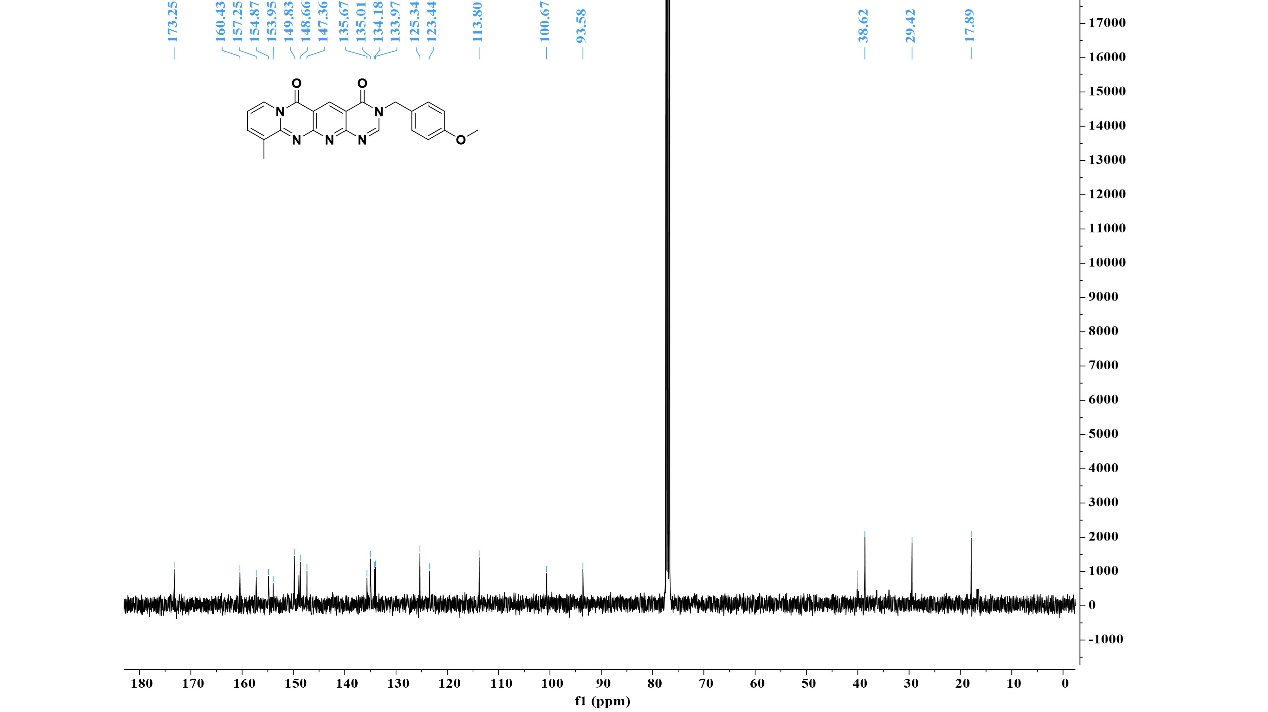


Figure S 26 ^13^C NMR (100 MHz, CDCl_3_) spectrum of compound 6g


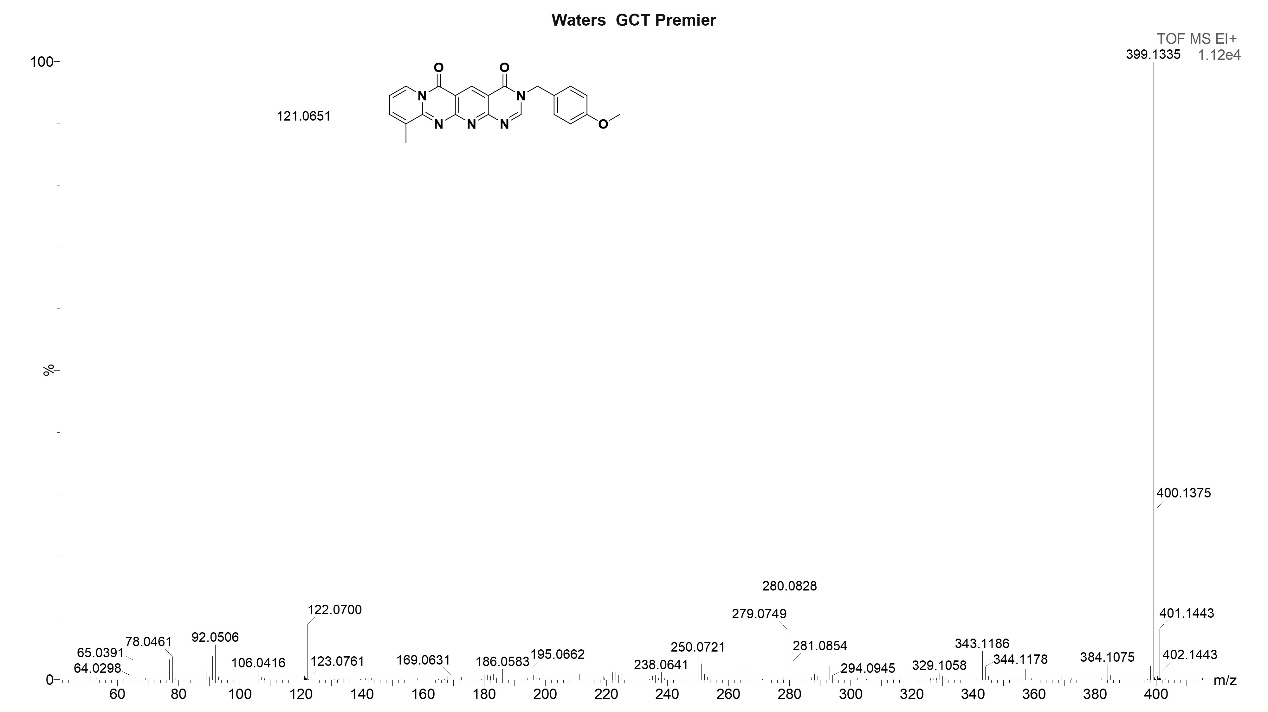


Figure S 27 HRMS of compound 6g


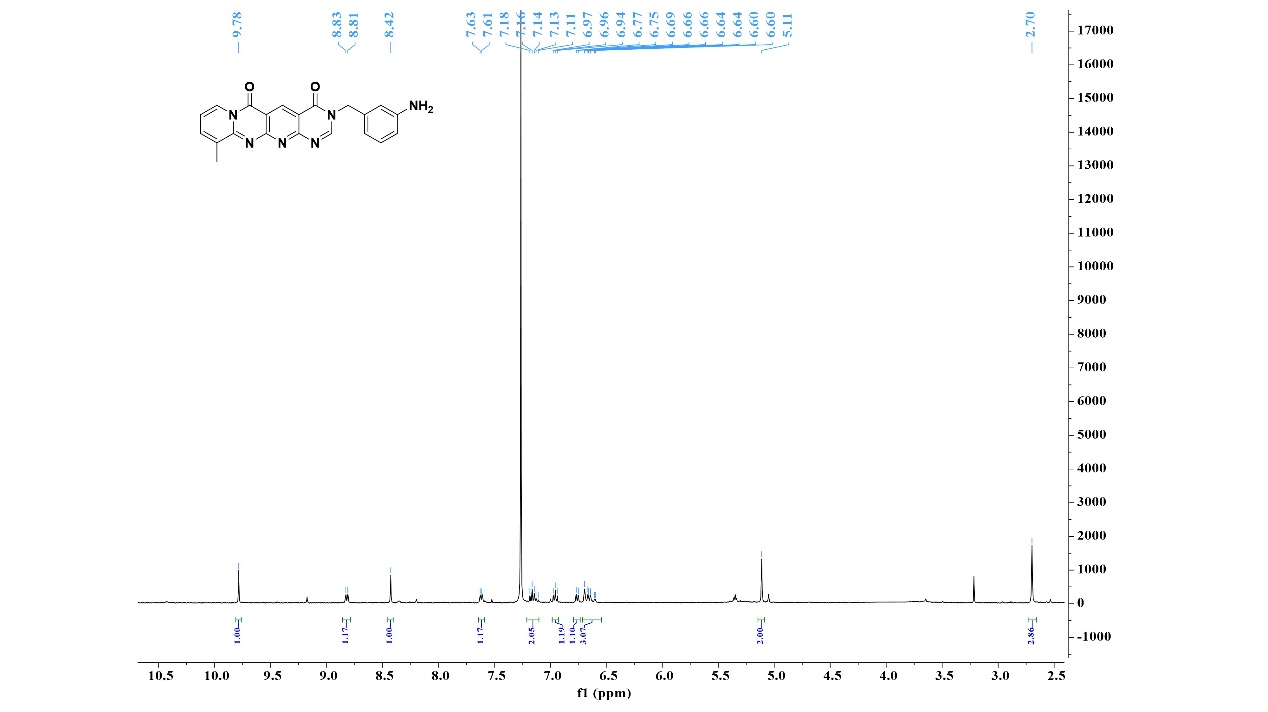


Figure S 28 ^1^H NMR (400 MHz, CDCl_3_) spectrum of compound 6h


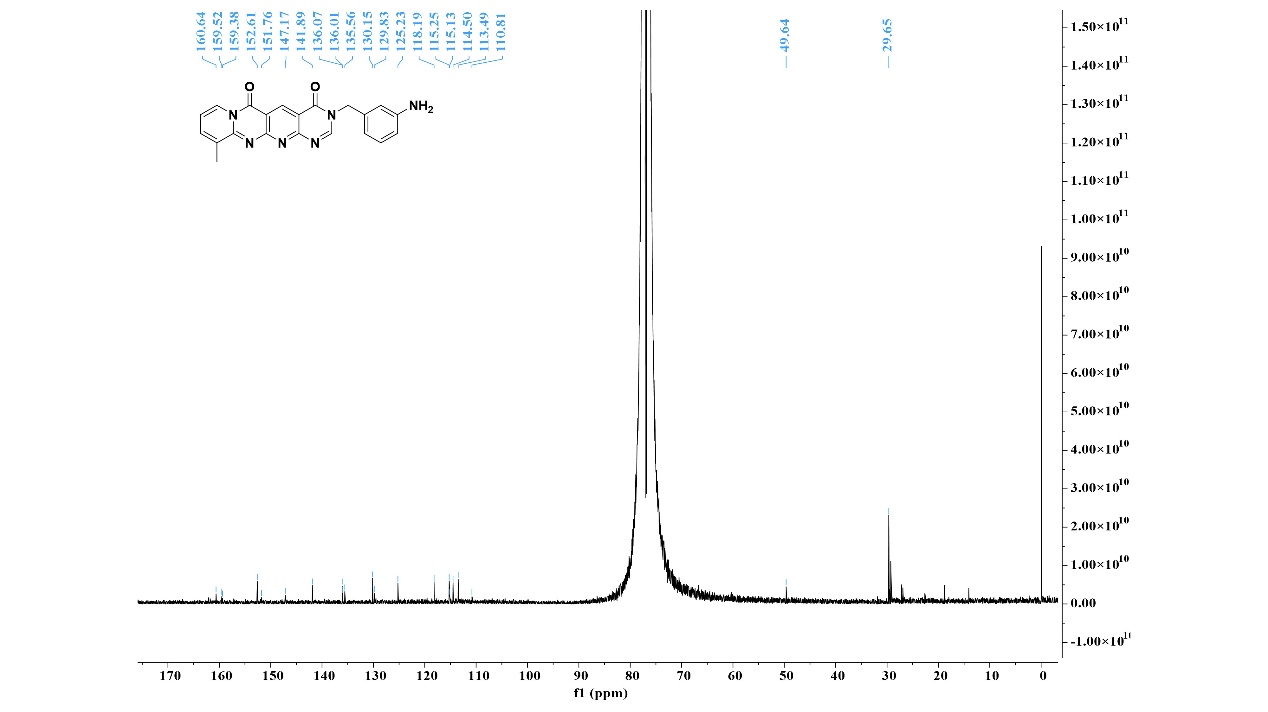


Figure S 29 ^13^C NMR (150 MHz, CDCl_3_) spectrum of compound 6h


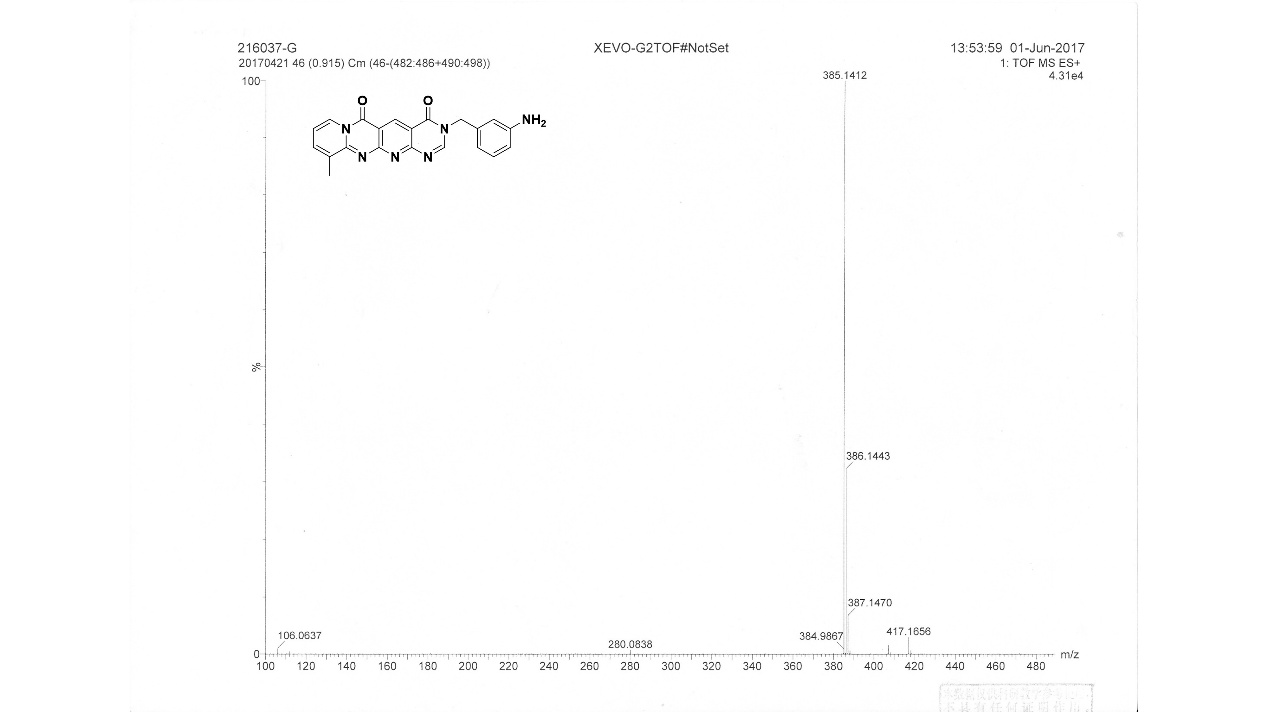


Figure S 30 HRMS of compound 6h


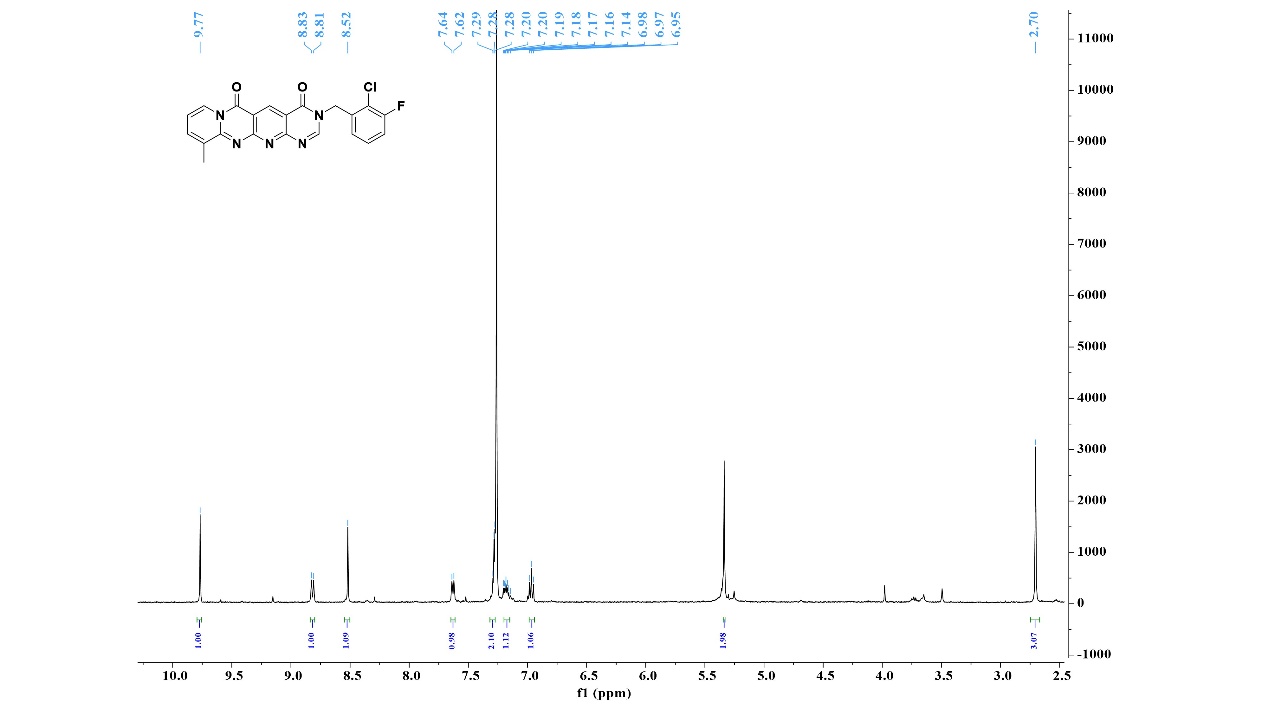


Figure S 31 ^1^H NMR (400 MHz, CDCl_3_) spectrum of compound 6i


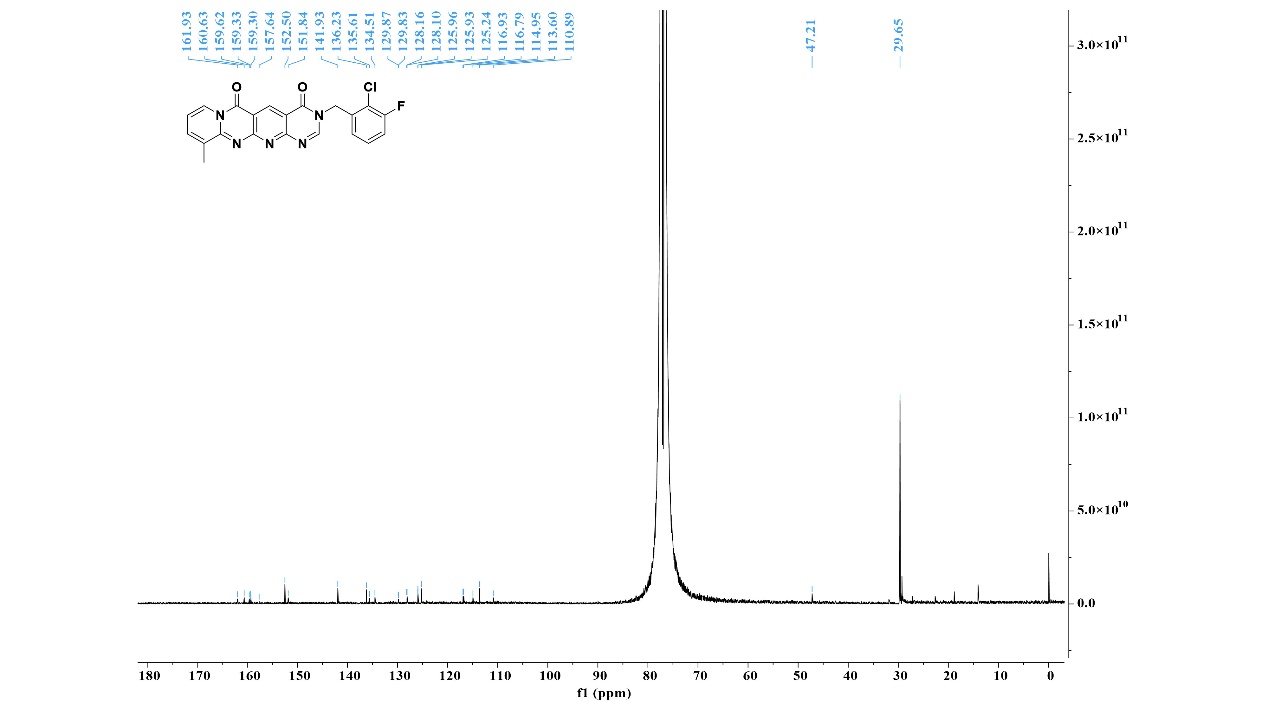


Figure S 32 ^13^C NMR (150 MHz, CDCl_3_) spectrum of compound 6i


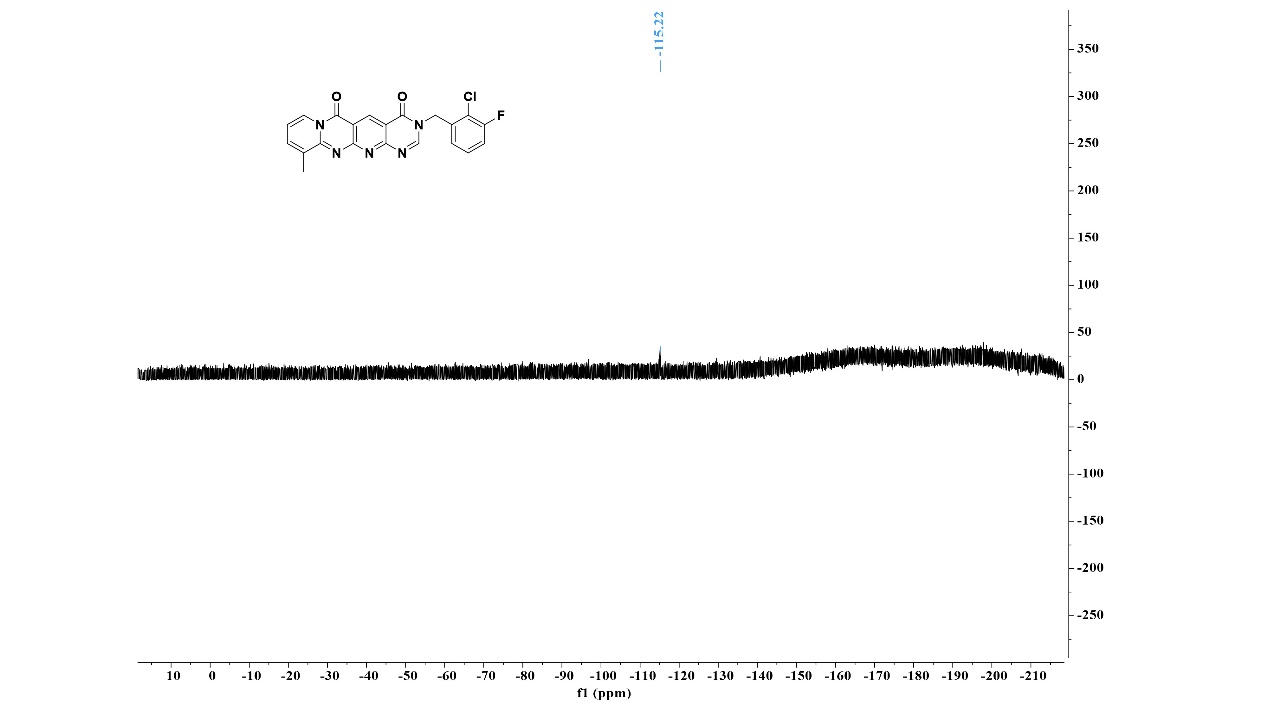


Figure S 33 ^19^F NMR (376 MHz, DMSO) spectrum of compound 6i


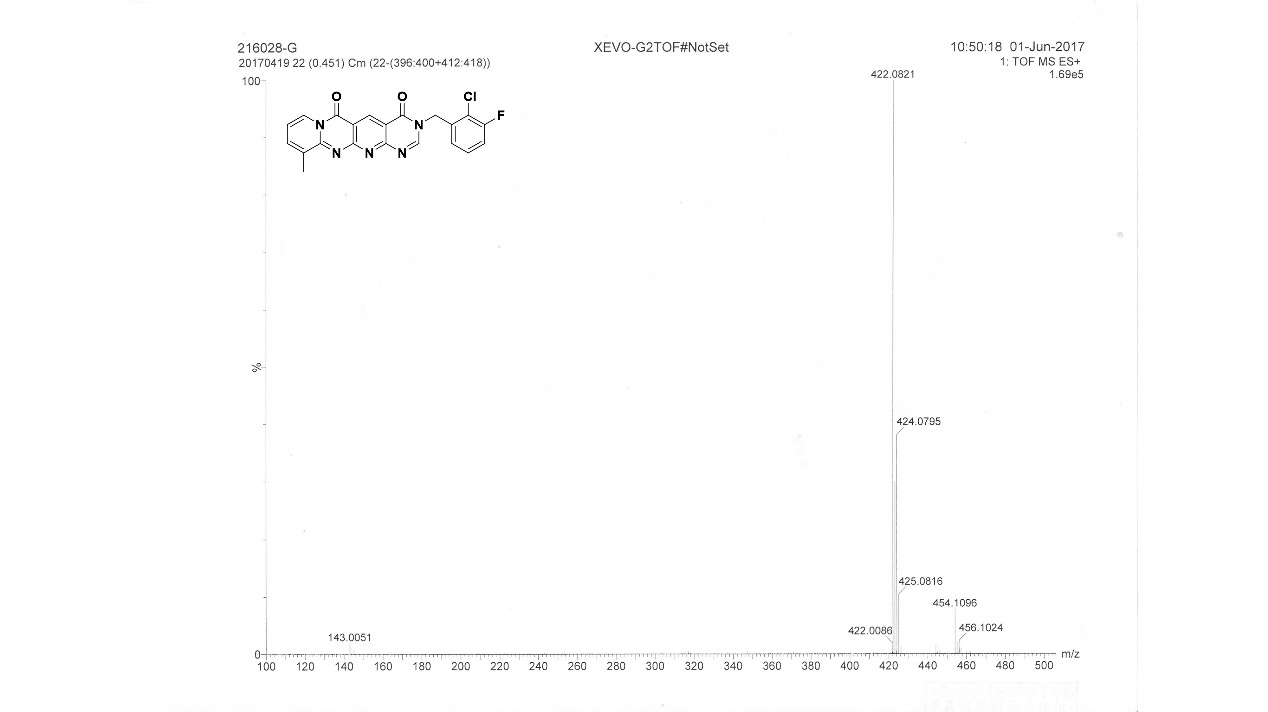


Figure S 34 HRMS of compound 6i


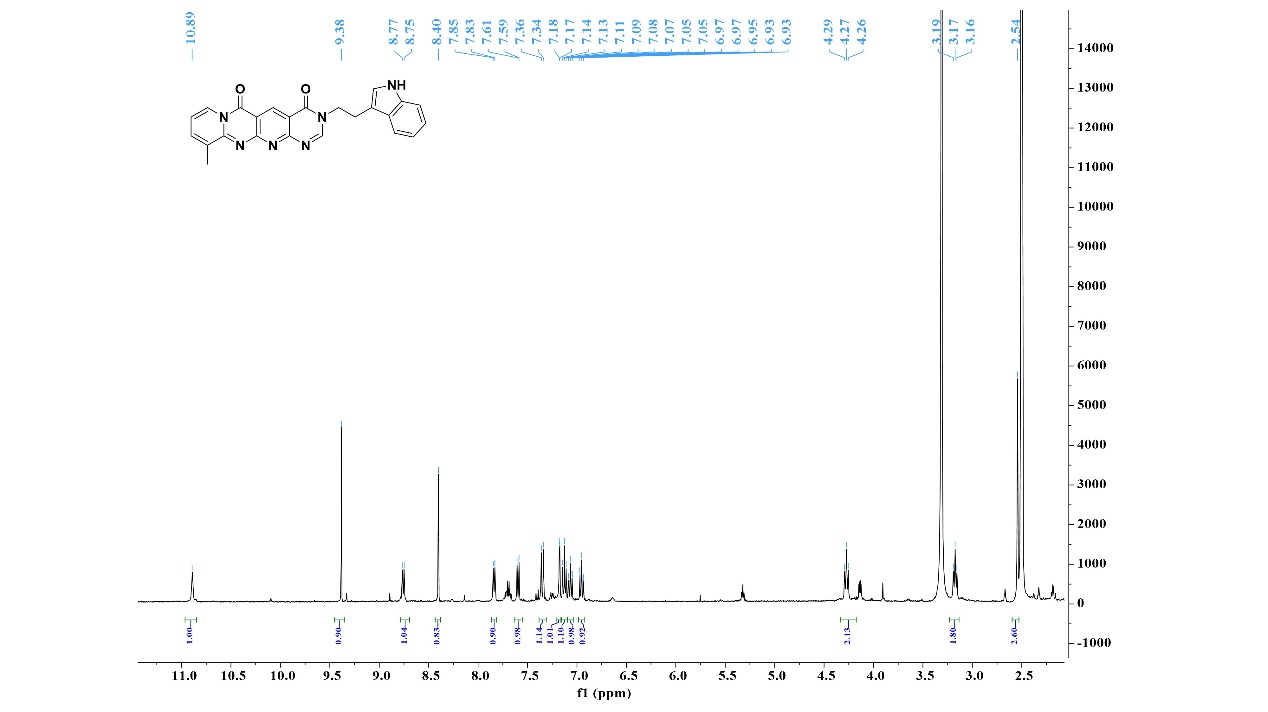


Figure S 35 ^1^H NMR (400 MHz, DMSO-*d*_6_) spectrum of compound 6j


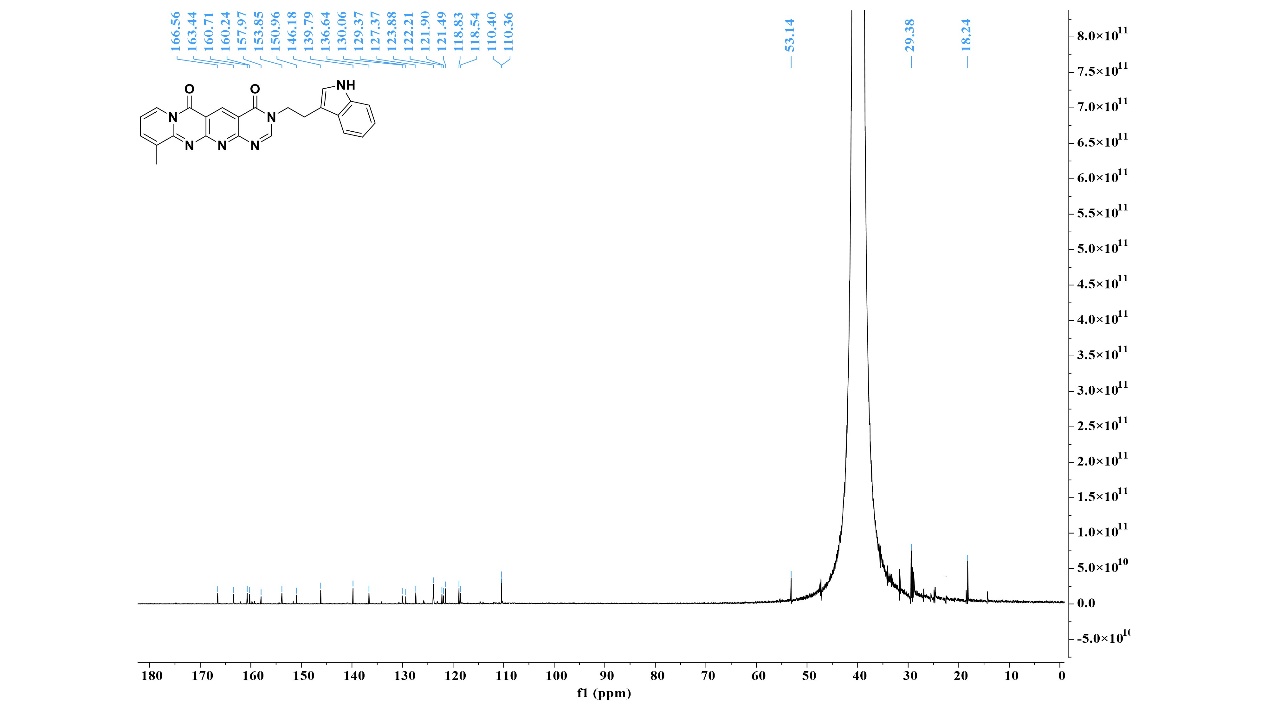


Figure S 36 ^13^C NMR (150 MHz, DMSO) spectrum of compound 6j


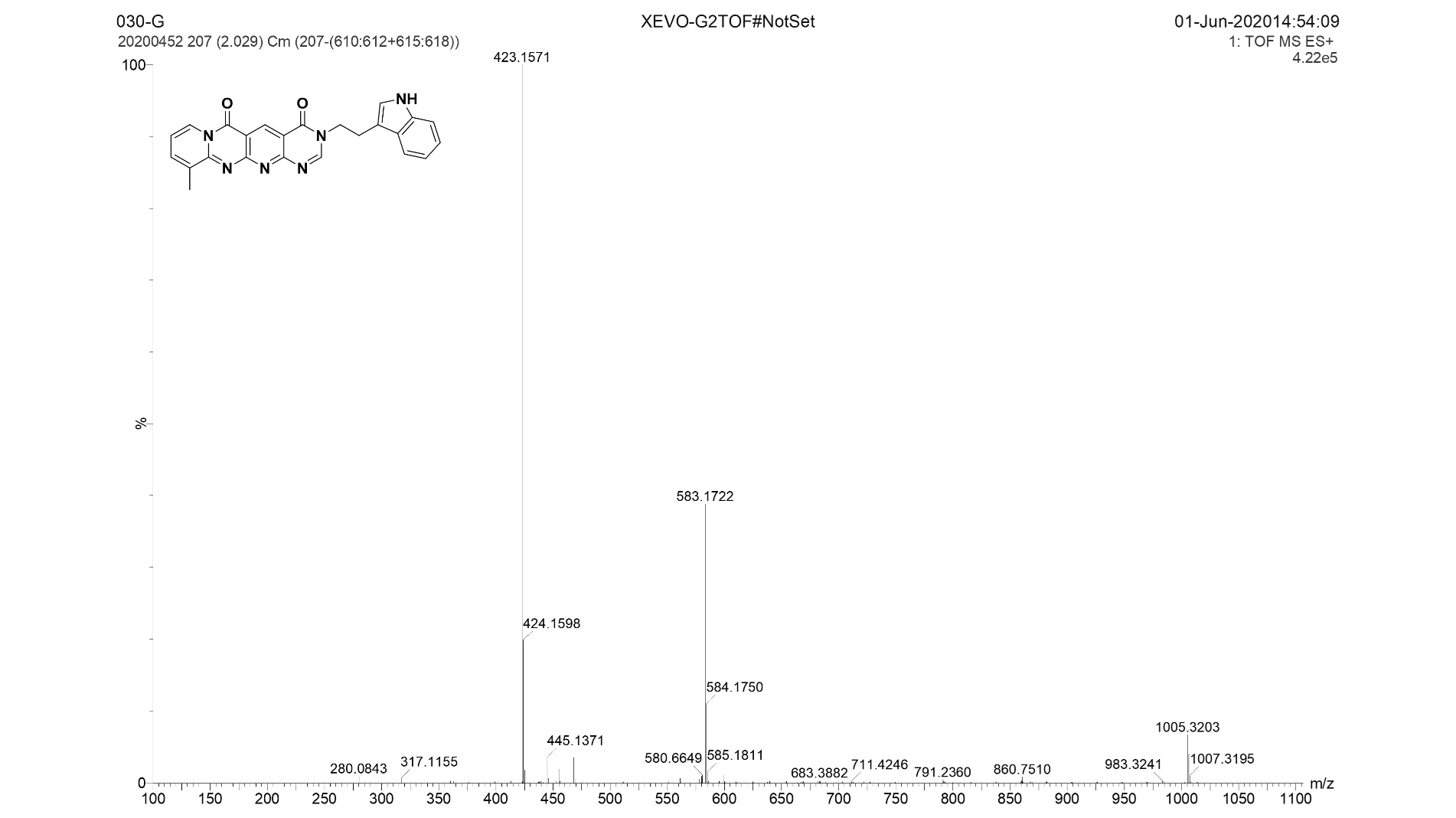


Figure S 37 HRMS of compound 6j


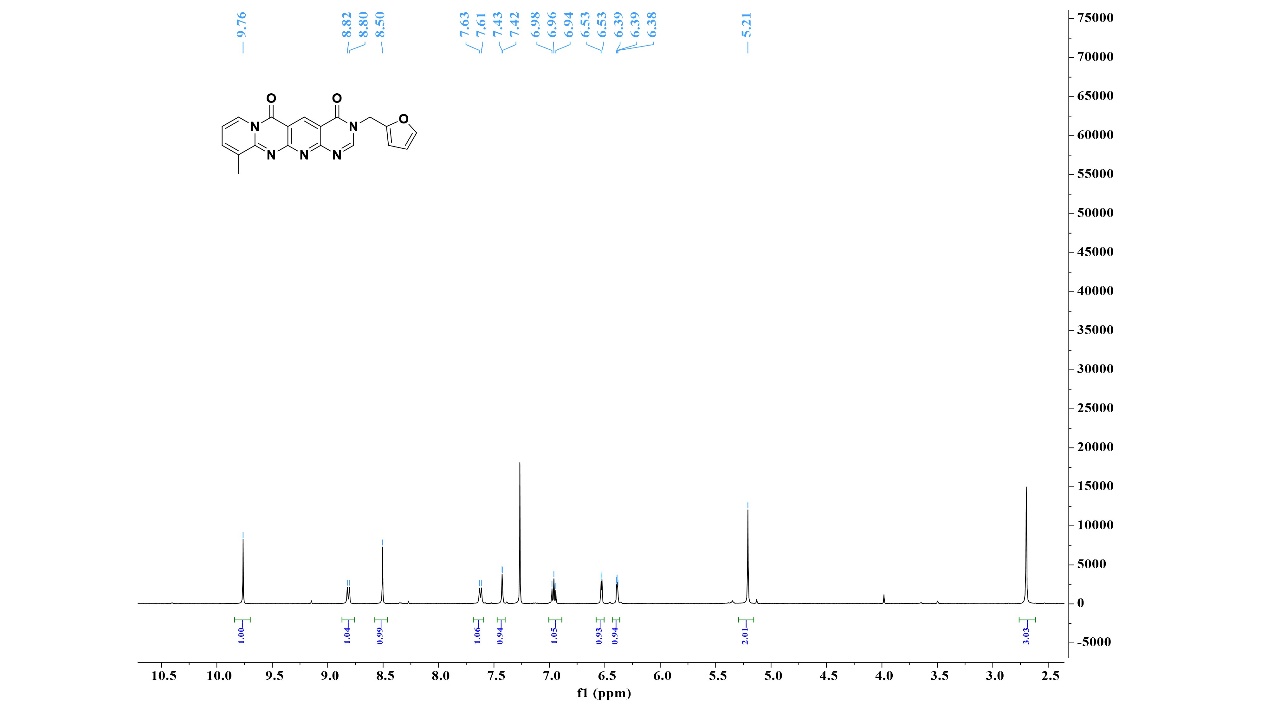


Figure S 38 ^1^H NMR (400 MHz, CDCl_3_) spectrum of compound 6k


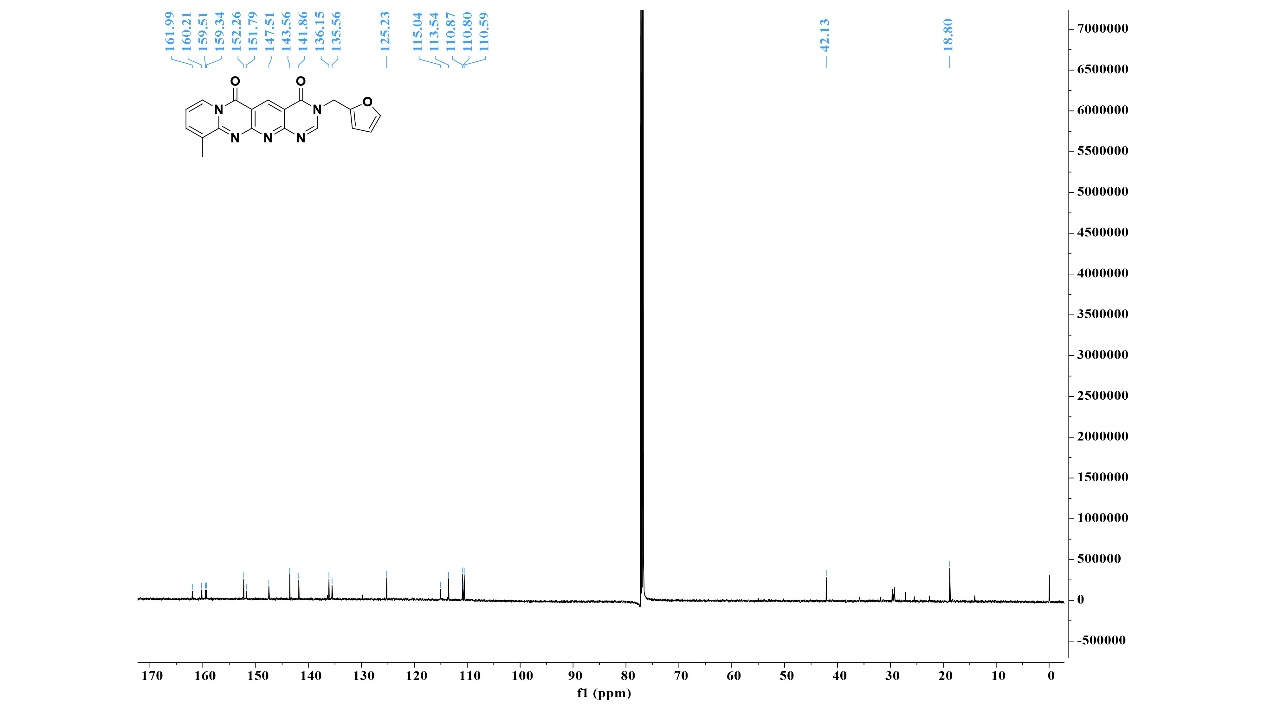


Figure S 39 ^13^C NMR (150 MHz, CDCl_3_) spectrum of compound 6k


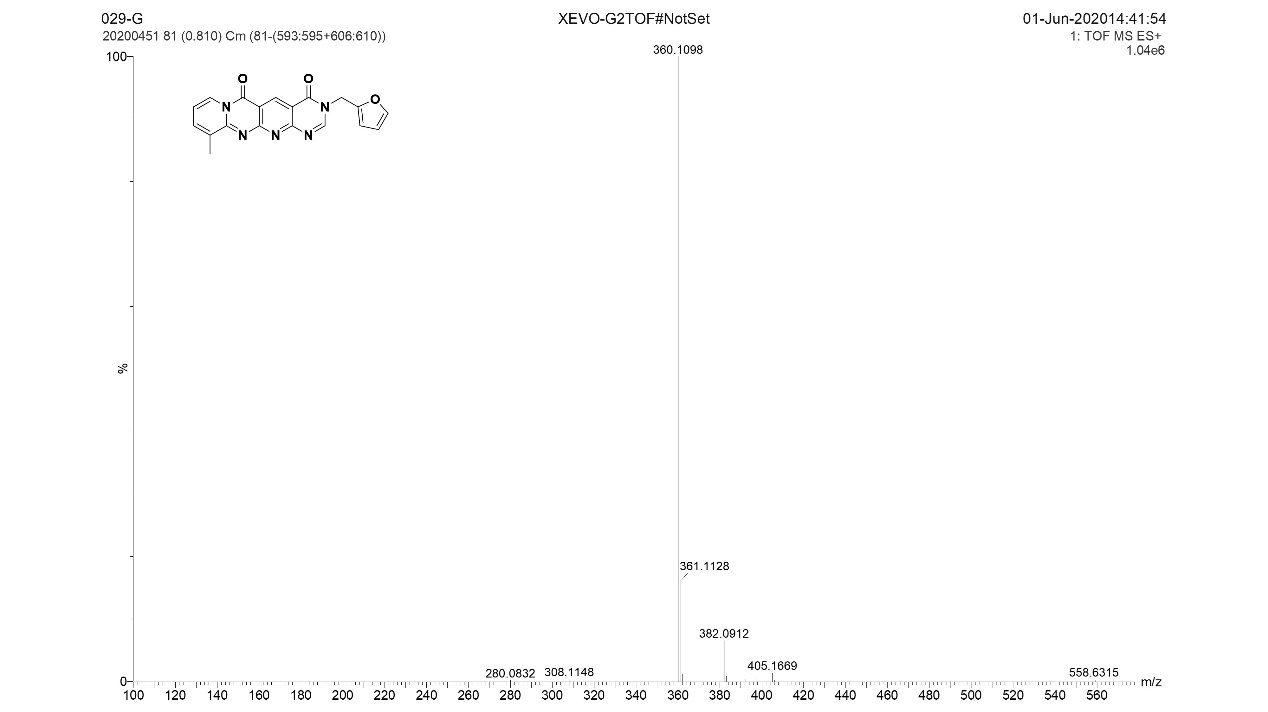


Figure S 40 HRMS of compound 6k


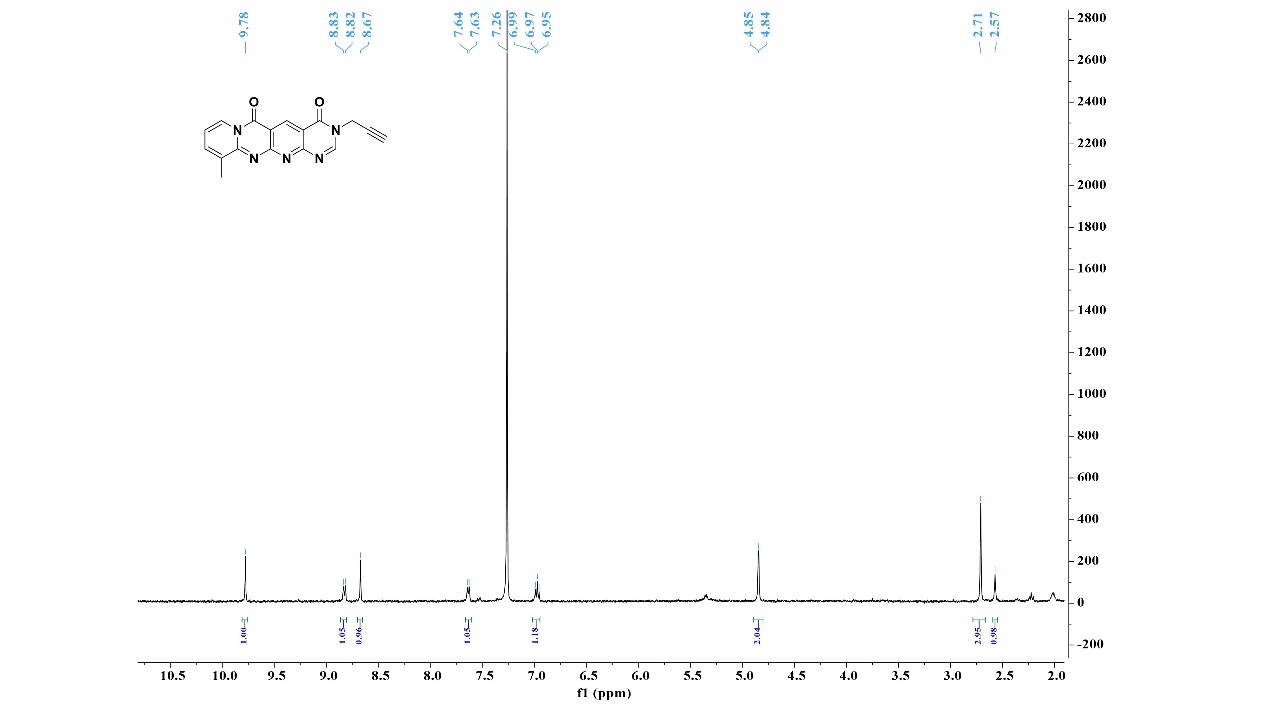


Figure S 41 ^1^H NMR (400 MHz, CDCl_3_) spectrum of compound 6l


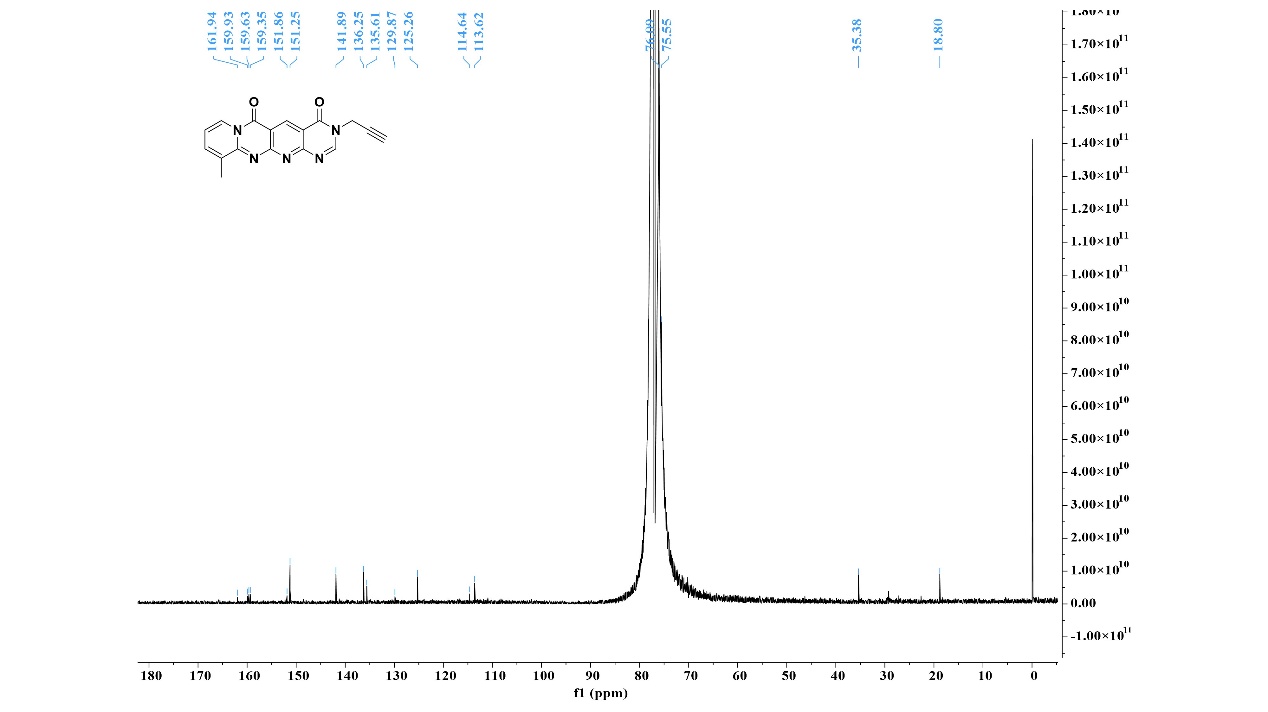


Figure S 42 ^13^C NMR (150 MHz, CDCl_3_) spectrum of compound 6l


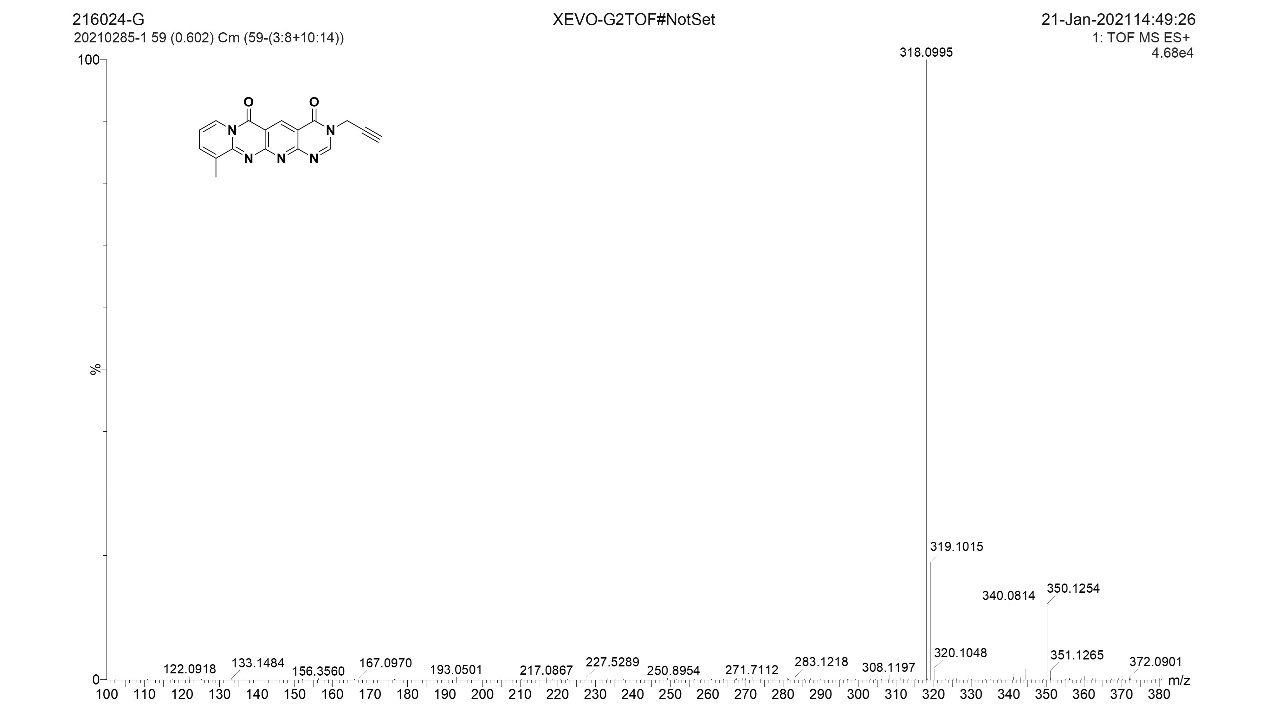


Figure S 43 HRMS of compound 6l
